# Macrophage-mediated trogocytosis contributes to destroying human schistosomes in a non-susceptible rodent host, *Microtus fortis*

**DOI:** 10.1038/s41421-023-00603-6

**Published:** 2023-10-05

**Authors:** Jia Shen, Siyu Zhao, Mei Peng, Yanguo Li, Lichao Zhang, Xiaoping Li, Yunyi Hu, Mingrou Wu, Suoyu Xiang, Xiaoying Wu, Jiahua Liu, Beibei Zhang, Zebin Chen, Datao Lin, Huanyao Liu, Wenyan Tang, Jun Chen, Xi Sun, Qi Liao, Geoff Hide, Zhijun Zhou, Zhao-Rong Lun, Zhongdao Wu

**Affiliations:** 1 Department of Parasitology, Zhongshan School of Medicine, Sun Yat-Sen University, Guangzhou, Guangdong, China; 2 Key Laboratory of Tropical Disease Control of the Ministry of Education, Sun Yat-sen University, Guangzhou, Guangdong China; 3 Provincial Engineering Technology Research Center for Biological Vector Control, Guangzhou, Guangdong China; 4 Institute of Drug Discovery Technology, School of Public Health, School of Medicine, Ningbo University, Ningbo, Zhejiang China; 5 Department of Hepatic Surgery and Liver Transplantation Center, Organ Transplantation Institute, The Third Affiliated Hospital, Sun Yat-sen University, Guangzhou, Guangdong China; 6 Hospital of Stomatology, Guanghua School of Stomatology, Sun Yat-sen University, Guangzhou, Guangdong China; 7 Department of Hepatic Surgery, The First Affiliated Hospital, Sun Yat-sen University, Guangzhou, Guangdong China; 8 Department of Immunology, Center for Precision Medicine and Engineering & Technology Research Center for Disease-Model Animals, Laboratory Animal Center, Zhongshan School of Medicine, Sun Yat-sen University, Guangzhou, Guangdong China; 9 Biomedical Research and Innovation Centre, School of Science, Engineering and Environment, University of Salford, Salford, UK; 10 Department of Laboratory Animals, Hunan Key Laboratory of Animal Models for Human Diseases, Central South University, Changsha, Hunan China; 11 State Key Laboratory of Biocontrol, School of Life Sciences, Sun Yat-Sen University, Guangzhou, Guangdong China

**Keywords:** Mechanisms of disease, Integrins, Innate immunity

## Abstract

*Schistosoma* parasites, causing schistosomiasis, exhibit typical host specificity in host preference. Many mammals, including humans, are susceptible to infection, while the widely distributed rodent, *Microtus fortis*, exhibits natural anti-schistosome characteristics. The mechanisms of host susceptibility remain poorly understood. Comparison of schistosome infection in *M. fortis* with the infection in laboratory mice (highly sensitive to infection) offers a good model system to investigate these mechanisms and to gain an insight into host specificity. In this study, we showed that large numbers of leukocytes attach to the surface of human schistosomes in *M. fortis* but not in mice. Single-cell RNA-sequencing analyses revealed that macrophages might be involved in the cell adhesion, and we further demonstrated that *M. fortis* macrophages could be mediated to attach and kill schistosomula with dependence on Complement component 3 (C3) and Complement receptor 3 (CR3). Importantly, we provided direct evidence that *M. fortis* macrophages could destroy schistosomula by trogocytosis, a previously undescribed mode for killing helminths. This process was regulated by Ca^2+^/NFAT signaling. These findings not only elucidate a novel anti-schistosome mechanism in *M. fortis* but also provide a better understanding of host parasite interactions, host specificity and the potential generation of novel strategies for schistosomiasis control.

## Introduction

Host specificity, defined as the range and diversity of host species that a parasitic organism is capable of infecting (or colonizing)^1^, is one of the most fundamental properties of parasites^2^. For example, *Schistosoma* parasites, causing schistosomiasis affecting more than 200 million people in 78 countries^3^, display different levels of adaptation (developmental states, survival time) and cause different degrees of pathogenicity in various definitive mammalian hosts^4–8^. *Schistosoma japonicum* is widely distributed in China, Indonesia and the Philippines. Humans and a number of wild and domestic animals (about 46 species), such as mice, dogs, goats, pigs, and cattle, are generally highly susceptible to this parasite. In these species, the worms develop well and cause severe lesions induced by a large number of egg granulomas in the liver and intestines which can even result in death^6,9^. Such animals are known as permissive hosts of *S. japonicum* and can serve as reservoirs for maintaining the transmission of this parasite in endemic regions, which in turn creates a significant obstacle to eliminating this pathogen^6^. However, some mammals, such as *Rattus norvegicus* and water buffalo, are referred to as semi-permissive hosts of *S. japonicum* in which only a few stunted worms are found after infection and produce very few eggs and slight damage to the liver^6,7^. Moreover, *Microtus fortis* (*M. fortis*), a species of vole (Reed Vole) that is widely distributed in Russia, Mongolia, Korea, and China^10^, has long been suggested as one of the hosts with the strongest resistance against *S. japonicum* infection, particularly in the epidemic regions of China^6,11^. It is defined as a non-permissive host. It has been reported that the development of *S. japonicum* in *M. fortis* is inhibited on day 12 after cercarial infection via skin penetration occurs, and the worms gradually shrink and ultimately die by 3 weeks post-infection^12^. During this period, the worms are always maintained in the schistosomula stage without the formation of reproductive systems and egg production. Intriguingly, this resistance is not affected by the environment (occurring in both wild and laboratory-bred animals) or geographical distribution of *M. fortis* and it has stable heritability^11,12^. Interestingly, in our study, we show that these innate anti-*Schistosoma* characteristics in *M. fortis* are also found to apply to another human infective species of Schistosoma, *S. mansoni*, which is widely distributed across Africa, the Middle East, the Central and South America. Host specificity in parasites is determined by the accumulation of historical parasite–host interactions and environmental forces^1^. Host specificity, as a topic of considerable interest in the field of disease ecology, has been extensively investigated for many decades, but the determinant factors have not yet been fully elucidated. Here, we consider that the high resistance of *M. fortis* against *S. japonicum* could be a valuable model system to understand host specificity and the mechanism of action against *Schistosoma* or other related pathogens.

Many previous studies have been carried out on the resistance mechanisms of *M. fortis* against schistosome (mainly on *S. japonicum*) infection over the last few decades. Jiang and his colleagues reported that the passive transfer of serum from *M. fortis* into infected mice could generate protective effects on recipient mice and result in a significant reduction in worm burden (36.2%)^13^. Further studies suggested that antibodies (e.g., IgG3), albumin, complement and cytokines in the serum might be the key factors involved in the resistance against schistosomes in *M. fortis*^14^. However, all these studies lacked in-depth functional verification. In addition, some effector molecules such as heat shock protein 90a (HSP90a)^15^, raryopherin a2^16^ and E77.43^17^ have also been reportedly involved in the schistosomula killing in *M. fortis*. Furthermore, microarray analysis comparing gene expression profiles between *M. fortis* and control permissive mice showed that some differentially expressed genes, such as microRNAs^18^, metabolites^19^ and hormones^20^, could be potentially involved in protective immunity. However, these results, again, lacked further verification with experiments. Recently, Li and colleagues reported that the immune responses in *M. fortis* were different from those in mice on the 10th day post-infection by using genome assembly and transcriptome analysis^21^. They showed that pathways of innate immunity were significantly enriched with upregulated genes in *M. fortis*, including leukocyte extravasation, antibody activation, Fc-gamma receptor-mediated phagocytosis and in the interferon signaling cascade pathway. Collectively, most of these published results are limited to the observation of changes in expression levels and histopathology but lack in-depth experimental verification due to the shortage of suitable research tools (e.g., commercial antibodies and transgenic animals). Thus far, the precise mechanism of intrinsic resistance against schistosomes and the factors that determine host specificity in *M. fortis* remain unknown.

In this study, we found a unique phenomenon associated with attached immune cells on the surface of schistosomula in *M. fortis* infected with *S. japonicum* and *S. mansoni*. We have provided unprecedented insight into the mechanism by which *M. fortis* macrophages kill the worm by means of contact-dependent trogocytosis. Furthermore, we demonstrated that this trogocytosis caused by macrophage was highly dependent on Complement C3 and its receptor CR3, while the activation of the Ca^2+^/NFAT pathway plays a key role in initiating trogocytosis-mediated killing. This work provides the first demonstration, to our knowledge, of a mammalian immune cell using trogocytosis to kill a multicellular helminth larva and provides mechanistic insights into macrophage cytotoxicity. This not only enriches an understanding of the anti-schistosome mechanism in *M. fortis* but also provides a better understanding of host–parasite interactions and host specificity.

## Results

### Large numbers of leukocytes were found to adhere to the surface of schistosomes in *M. fortis*

**Fig. 1:**
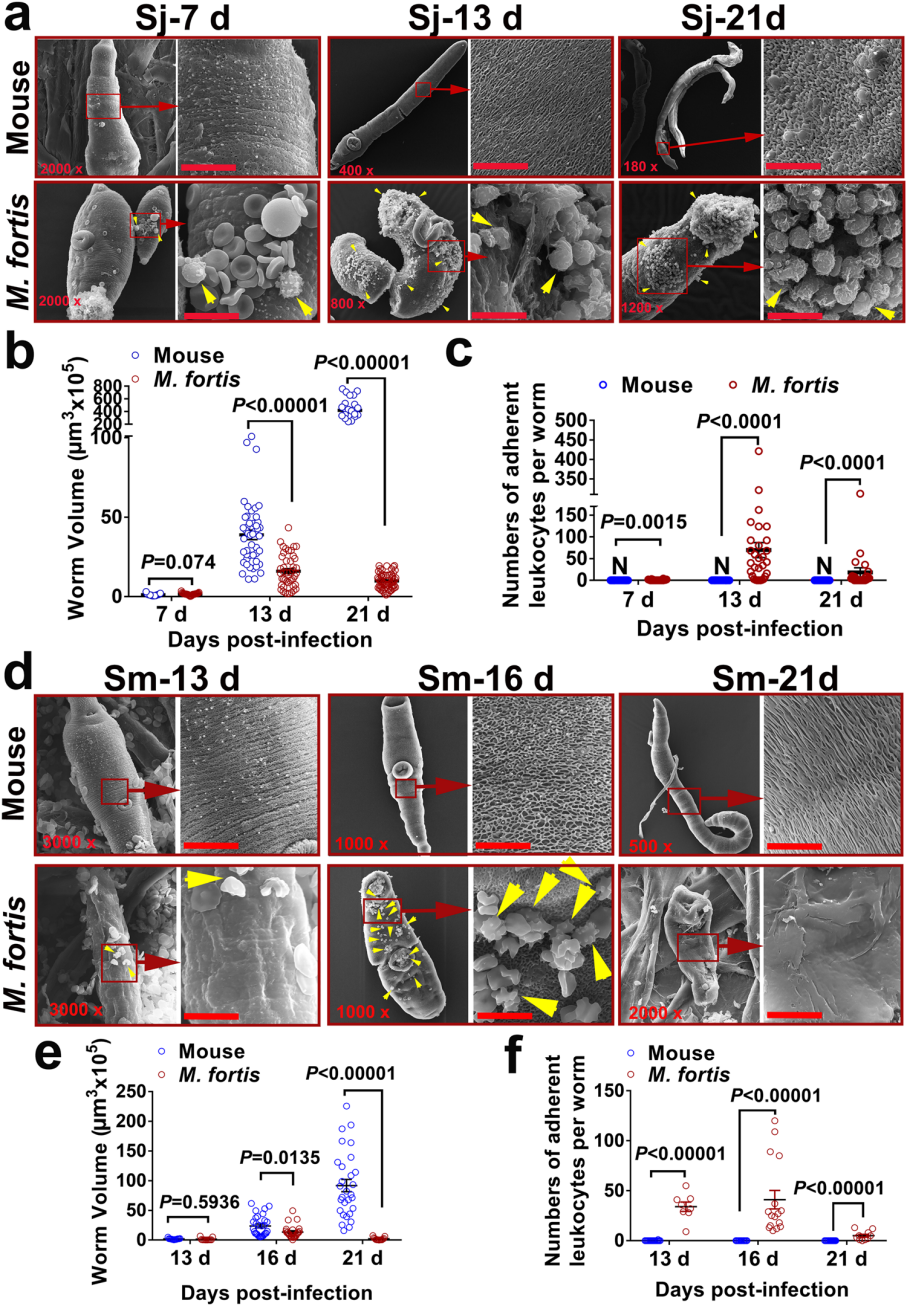
A large number of leukocytes adhere to the schistosomula of *S. japonicum* and *S. mansoni* in *M. fortis*. **a** Representative electron micrographs of *S. japonicum* collected from infected mice (*Mus musculus*, BALB/c) and *M. fortis* at 7, 13 and 21 days post-infection. The yellow arrows indicate the host cells attached to *S. japonicum*. Sj, *S. japonicum*. Scale bars, 10 μm. **b** Comparison of the size of *S. japonicum* in mice and *M. fortis* at 7, 13, and 21 days post-infection. Mean values are represented by horizontal bars. **c** Number of leukocytes adhering to *S. japonicum* that can be observed under a scanning electron microscope. N, nondetectable. **d** Representative electron micrographs of *S. mansoni* collected from infected mice (*Mus musculus*, BALB/c) and *M. fortis* at 13, 16 and 21 days post-infection. The yellow arrows indicate the cell adherence to *S. mansoni*. Sm, *S. mansoni*. Scale bars, 10 μm. **e**, **f** Quantitative analysis of the size of *S. mansoni* (**e**) and the numbers of leukocytes attached to *S. mansoni* (**f**) in mice and *M. fortis* at 13, 16, and 21 days post-infection. Groups of 5 mice or *M. fortis* were used for each experimental condition. The data are expressed as the mean ± SEM. Similar results were obtained in more than three repeated experiments.

We first compared the development of *S. japonicum* in mice (*Mus musculus*, BALB/c) and *M. fortis* using scanning electron microscopy (SEM). As shown in Fig. 1a, b, the size of *S. japonicum* in *M. fortis* was much smaller than that in mice after 7 days post-infection (*P* < 0.0001) (Fig. 1b). In addition, compared with the increasing size of the schistosomes in mice along with the infection time, the worm development in *M. fortis* was hindered after 13 days post-infection (Fig. 1a, b). Interestingly, we found that large numbers of leukocyte-like cells with microvilli and protrusions (Supplementary Fig. S1), which were different from the smooth and flat biconcave disc-shaped morphology of erythrocytes (Supplementary Fig. S1), adhered to the surface of *S. japonicum* in *M. fortis*, and the number of adherent leukocytes peaked on 13 days post-infection (Fig. 1a, c). However, no leukocyte attachment on any *S. japonicum* was found in mice at any time post-infection (Fig. 1a, c). Importantly, a similar phenomenon was also found for *S. mansoni* infection in *M. fortis* (Fig. 1d–f). The development of *S. mansoni* was blocked at 16 days post-infection when the number of attached host cells peaked, and all worms were eliminated at 22 days after infection (Fig. 1d–f). These results suggest that the adherence of leukocytes to schistosomula may be involved in the resistance to schistosomes infection in *M. fortis*.

### Single-cell RNA-sequencing analysis of the adherent leukocytes on schistosomes in *M. fortis*

**Fig. 2:**
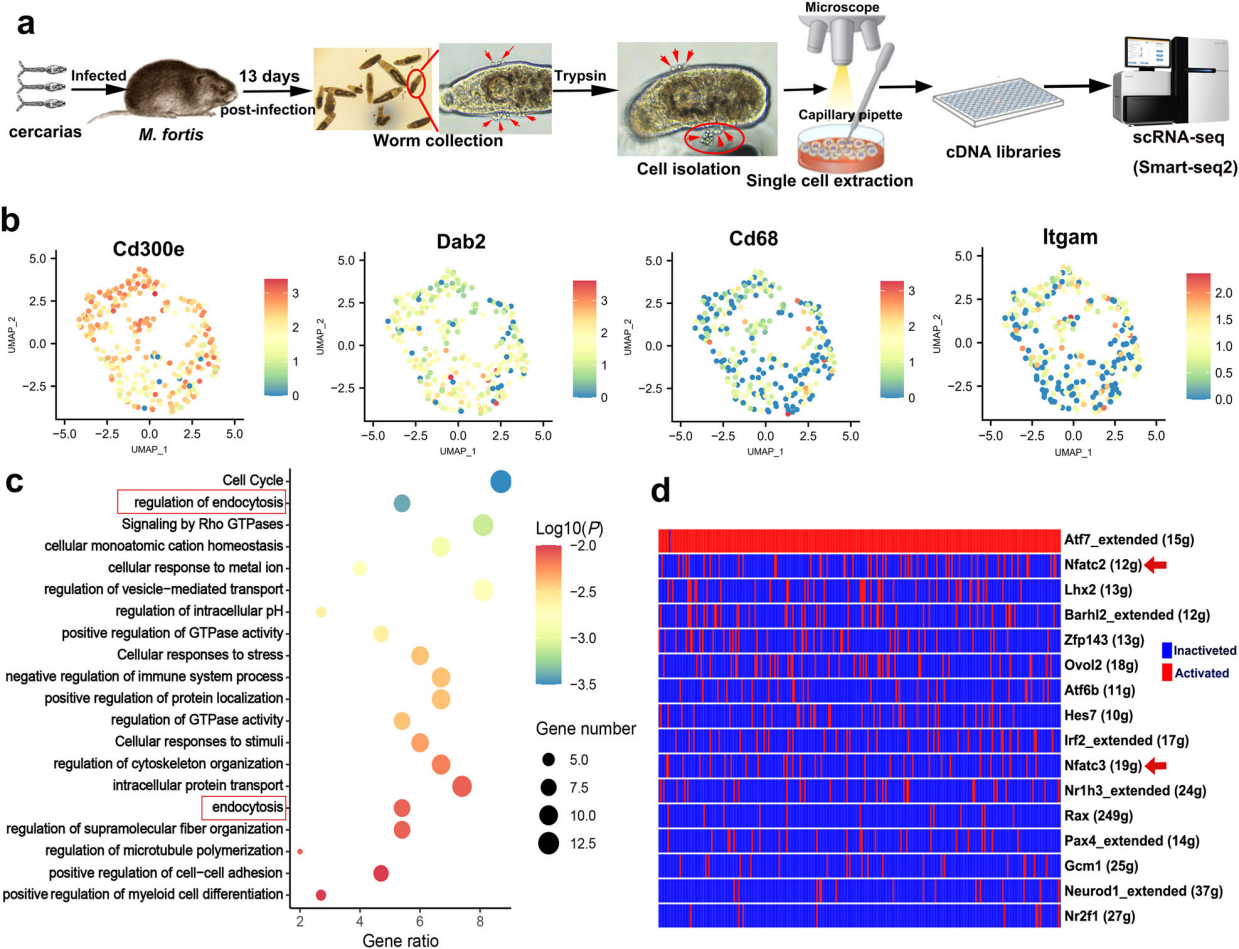
Single-cell RNA-sequencing analyses identify the adherent leukocytes on schistosomes in *M. fortis*. **a** Overview of the scRNA-seq analyses, illustrating the isolation and scRNA-seq analysis of the leukocytes adhered to the surface of schistosomes in *M. fortis***. b** UMAP maps indicate the expression of canonical macrophage markers. Data are shown as normalized transcript counts on a color-coded logarithmic scale. **c** Gene set enrichment analysis of the top 200 highly expressed genes. **d** Heatmap showing the regulons (rows) activity in each cell (columns) as estimated by SCENIC. For each regulon, the transcription factor and the number of predicted target genes are indicated. The gene name suffix (_extended) represents that the reliability of the annotated target gene is relatively low.

To identify the cell identity of the adhered leukocytes on the surface of *S. japonicum* in *M. fortis*, the worms were collected from infected *M. fortis* at 13 days post-infection, and the adhered cells were separated by trypsin. After sorting the live single cells into 96-well plates, single-cell RNA-sequencing (scRNA-seq) was performed using SmartSeq2^22^. Figure 2a provides a summary diagram of the cell isolation and scRNA-seq. After obtaining the clean reads of *M. fortis* by removing the reads from *S. japonicum*, dimension reduction and clustering were employed to visualize the individual transcriptomes of all cells in the unified dataset (Fig. 2b). Independently, unsupervised shared nearest neighbor clustering was performed, which showed that these cells had no obvious clustering characteristics based on their transcriptomic profiles (Fig. 2b). Cluster annotation was guided by the expression of canonical cell markers, as exemplified in Fig. 2b. Based on the expression profiles, this group of cells was identified as macrophages (Fig. 2b). Furthermore, gene set enrichment analysis of the top 200 highly expressed genes showed that the adherent cells exhibited higher activity in the “endocytosis” pathway and some pathways involved in the phagocytic process^23–25^, such as “Signaling by Rho GTPases”, “regulation of cytoskeleton organization” and “regulation of supramolecular fiber organization” (Fig. 2c). These results suggest that macrophages may be involved in the adhesion to schistosomes in *M. fortis*. Transcription factors (TFs) play a key role in regulating the progress of cellular biological behavior and signal pathways. To screen key TFs, the regulatory network analysis of scRNA-seq was performed by SCENIC^26^. The result showed that the nuclear factor of activated T cells (NFAT) family (NFATc2 and NFATc3) was in the top list of activated TFs (Fig. 2d), suggesting that NFAT might play a key role in regulating the process of cell adhesion-mediated effects in *M. fortis*. Taken together, the scRNA-seq analyses identify the possible signaling and transcriptional regulators involved in cell adhesion and subsequent effects in *M. fortis*.

### *M. fortis* macrophages are involved in mediating adherence and killing of *S. japonicum* in vitro and in vivo

**Fig. 3:**
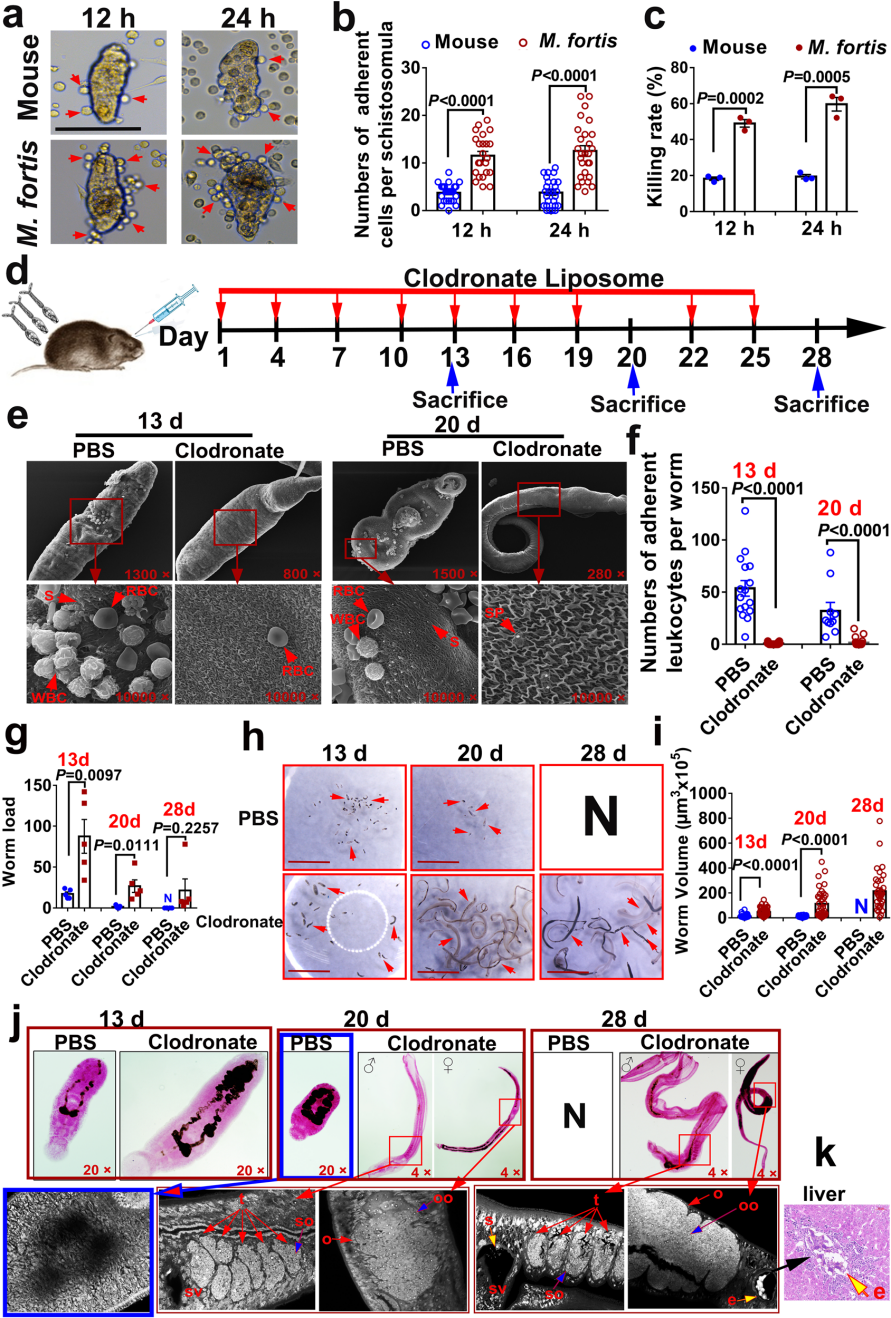
*M. fortis* macrophages are involved in mediating adherence and killing of schistosomes in vitro and in vivo. **a** Representative images of mouse and *M. fortis* macrophages adhering to schistosomula of *S. japonicum* after incubation for 12 and 24 h in RPMI-1640 medium supplemented with 10% non-heat-inactivated FBS in vitro. The arrows indicate macrophages adhering to schistosomula. Scale bars, 100 μm. **b**, **c** The number of cells adhering to *S. japonicum* schistosomula (**b**) and the schistosomula killing rates (**c**) after co-culture with macrophages from BALB/c mice and *M. fortis* for 12 and 24 h. The data are expressed as the mean ± SEM of three animals per group. Data are representative of three independent experiments. **d** Schematic illustration of the clodronate liposome-mediated macrophage depletion protocol. **e** Representative electron micrographs of leukocyte attachment on *S. japonicum* in *M. fortis* treated with PBS or clodronate at 13- and 20-day post-infection. RBC red blood cell, WBC white blood cell, S spine, SP sensory papillae. **f** Number of leukocytes adhering to *S. japonicum* that can be observed under SEM. **g** Worm load in *M. fortis* treated with PBS- or clodronate- liposomes at 13-, 20- and 28-day post-infection. The data are expressed as the mean ± SEM of five animals per group. **h** Micrographs of representative *S. japonicum* from *M. fortis* treated with PBS- or clodronate- liposome. N, no worms were found (eliminated by the host). Scale bars, 5 mm. **i** Comparison of the size of *S. japonicum* at the indicated times post-infection in *M. fortis* treated with PBS or clodronate. **j** Morphological analysis of the reproductive organs of *S. japonicum* at 13-, 20- and 28-day post-infection in *M. fortis* treated with PBS or clodronate. The worms were stained with hydrochloric carmine and observed under a light microscope (Upper) and confocal laser scanning microscopy (Lower). ♂, male worms; ♀, female worms. t, testicular lobules; so, spermatocytes; sv, seminal vesicle; o, ovary; oo, oocytes; s, sperm; e, egg. N, none detected. **k** Eggs of *S. japonicum* deposited in the liver of 1 out of 10 *M. fortis* treated with clodronate. The yellow arrow indicates the eggs of *S. japonicum*. *n* = 5 *M. fortis* per group. Data shown are mean ± SEM and repeated twice with similar results.

To examine the effect of *M. fortis* macrophages that were bound to schistosomula and to investigate the correlation between cell attachment and cytotoxicity of the schistosomula, we first compared the ability of macrophages from BALB/c mice and *M. fortis* to adhere to and kill the schistosomula in vitro. As shown in Fig. 3a, b and Supplementary Video S1, few or no murine macrophages could be observed that adhered to the schistosomula of *S. japonicum* after being co-cultured for 12 and 24 h in RPMI-1640 medium supplemented with 10% non-heat-inactivated fetal bovine serum (FBS). In contrast, a large number of *M. fortis* macrophages were found to adhere to schistosomula at the same time points following co-culture (Fig. 3a, b and Supplementary Video S2). In addition, *M. fortis* macrophages were observed to kill significantly more schistosomula of *S. japonicum* than murine macrophages at 12 and 24 h following co-culture (*P* < 0.05) (Fig. 3c). Supplementary Video S2 showed the process of *M. fortis* macrophage attaching to the surface of schistosomula, followed by a decrease in worm activity and eventual death (immobility). Similar results were also found in co-cultures of macrophages with schistosomula of *S. mansoni* (Supplementary Fig. S2a–c). These results suggest that *M. fortis* macrophages naturally possess the ability to adhere to the schistosomula of both species, and it seems that the increased adherence of host macrophages to the larvae correlated with a high killing rate of these worms.

To further study the role of macrophages in the defense against schistosome infection in vivo, infected *M. fortis* underwent macrophage depletion via intraperitoneal (i.p.) administration of clodronate liposomes^27^ (Fig. 3d). Consistent with prior studies^28^, flow cytometric analysis revealed a significant reduction in the total CD11b^+^ population in the liver of *M. fortis* following clodronate liposome treatment when compared with control PBS-treated liposomes (Supplementary Fig. S2d, e), which suggests that macrophages were efficiently depleted in *M. fortis*. When adherent leukocytes were compared, no or few leukocyte adherence could be observed on the worms when macrophages were depleted with clodronate liposomes (Fig. 3e, f). Furthermore, the depletion of macrophages resulted in significant decreases in resistance to *S. japonicum* infection in *M. fortis*, which were characterized by a significant increase in worm load (Fig. 3g), increase in the development of worms (Fig. 3e, h, i). Intriguingly, compared with the schistosomulum status in the control group, some schistosomes in the macrophage-depleted group could be mature and reproducing (Fig. 3j, k). As shown in Fig. 3j, some *S. japonicum* collected from the infected animals treated with clodronate liposome were found to develop into females and males at 20 days (about 16.6%) and 28 days (about 10%) post-infection. In males, we observed six to eight testicular lobules containing large amounts of spermatogonia and spermatocytes (Fig. 3j). Furthermore, in 20-day-old *S. japonicum*, the structure of the seminal vesicle was observed, while later (28-day-old *S. japonicum*) large numbers of small sperm were found in the seminal vesicle. In females, the ovary was composed of abundant oogonia and oocytes, and the uterine structures were clearly observed in 20-day-old *S. japonicum* (Fig. 3j). Later, unreleased eggs were found in the uterus in the 28-day-old female *S. japonicum* and eggs produced by pairs were found deposited in the liver of 1 out of 10 *M. fortis* treated with clodronate (Fig. 3k). However, no obvious reproductive organs were ever observed in the *S. japonicum* collected from *M. fortis* treated with PBS liposome (Fig. 3j). Together, these data suggest that macrophages were involved in the defense against schistosome infection by mediating immune cell adhesion to the schistosome in *M. fortis*.

### *M. fortis* Complement C3 and its receptor CR3-mediated macrophage adherence and killing of *S. japonicum*

**Fig. 4:**
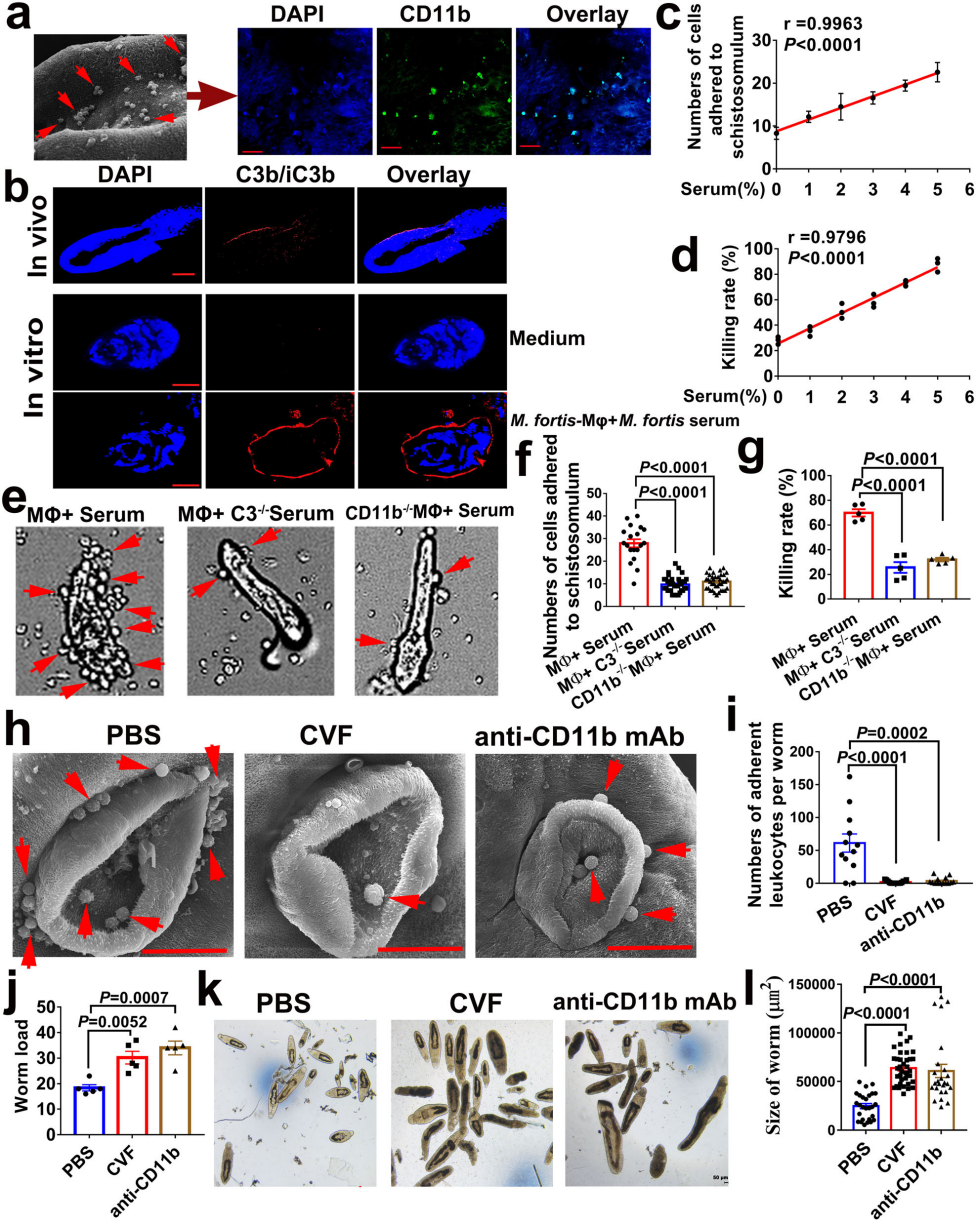
*M. fortis* macrophage-mediated adherence and killing of *S. japonicum* required Complement C3 and its receptor CR3. **a** Immunofluorescence detected CD11b expression (green) of the adherent leukocytes on the surface of schistosomula in *M. fortis*, and the nucleus was stained with DAPI (blue). Scale bars, 20 μm. The image on the left: SEM shows the profile of the adherent leukocytes on the surface of schistosomula in *M. fortis*. Scale bars, 50 μm. **b** Immunofluorescence detected the binding of C3b/iC3b (red) to the surface of *S. japonicum* collected from *M. fortis* on 13 days after infection (in vivo, scale bars, 50 μm) and the schistosomula co-cultured with *M. fortis* macrophages in the presence of 5% *M. fortis* serum or culture medium alone in vitro (scale bars, 20 μm). The nucleus was stained with DAPI (blue). **c** Pearson correlation analysis of the correlation between the concentration of normal *M. fortis* serum (%) in the RPMI-1640 medium (supplemented with 10% heat-inactivated FBS to eliminate the interference factor of complements in the culture medium) and the numbers of *M. fortis* macrophages adhered to a schistosomula after co-culturing for 12 h in vitro (*r* = 0.9963; *P* < 0.0001). Three *M. fortis* were used and repeated thrice with similar results. **d** Pearson correlation analysis of the correlation between the concentration of normal *M. fortis* serum (%) in the RPMI-1640 medium (supplemented with 10% heat-inactivated FBS) and the killing rate of *M. fortis* macrophages against schistosomula after co-culturing for 12 h in vitro (*r* = 0.9796; *P* < 0.0001). Three *M. fortis* were used and repeated thrice with similar results. **e** Representative micrographs of macrophage adherence to schistosomula after incubation of the schistosomula with *M. fortis* macrophages for 12 h in RPMI-1640 medium (supplemented with 10% heat-inactivated FBS) in the presence of 5% *M. fortis* serum. MΦ + Serum: macrophages cultured with schistosomula in the presence of normal *M. fortis* serum; MΦ+C3^*−/−*^ Serum: macrophages cultured with schistosomula in the presence of C3-inactivated serum of *M. fortis* by cobra venom factor (CVF) treatment; CD11b^*−/−*^ MΦ + Serum: macrophages cultured with schistosomula in the presence of normal *M. fortis* serum where the CD11b function was blocked by CD11b mAb. The arrows indicate macrophage adherence to schistosomula. **f**, **g** Quantitative analysis of the numbers of cells attached to schistosomula (**f**) and the schistosomula killing rates (**g**) after depletion of C3 in *M. fortis* serum and blocking of CD11b function of *M. fortis* macrophages in vitro. Three *M. fortis* were used and repeated thrice with similar results. **h**–**l** CVF and anti-CD11b mAb were respectively injected into infected *M. fortis* to deplete C3 and block CD11b function in vivo. On day 13 after infection, the leukocytes’ attachment to the surface of *S. japonicum* (**h**, **i**), the worm load (**j**) and the development of *S. japonicum* (**k**, **l**) were compared. The arrows indicate leukocyte adherence to schistosomes. All data are expressed as the mean ± SEM of 5 animals per group. Similar results were obtained in three repeated experiments.

We next investigated the key adhesion molecule and related pathways mediating the cell attachment and subsequent schistosome killing in *M. fortis*. Previous studies reported that leukocyte adhesion and migration were mainly mediated by integrins, such as CD11a/CD18, CD11b/CD18 (Mac-1) and CD11c/CD18, alongside the intercellular adhesion molecule (ICAM1)^29–31^. We initially analyzed the expression of related adhesion molecules in the scRNA-seq and found that the expression of CD11b (*Itgam*) was the most abundant (Supplementary Fig. S3a). In addition, immunofluorescence analysis showed that the majority of adherent cells on the surface of 13-day *S. japonicum* in *M. fortis* were CD11b-positive cells (Fig. 4a). CD11b/CD18, also named the complement receptor (CR) 3, is one of the receptors of complement C3, which is known to bind the C3 fragment iC3b leading to leukocyte trafficking and migration, adhesion, synapse formation, and phagocytosis^31^. Interestingly, immunofluorescence analysis showed that C3b/iC3b signals were present on the surface of 13-day *S. japonicum* in *M. fortis* and the schistosomula co-cultured with *M. fortis* macrophages in the presence of 5% *M. fortis* serum in vitro (Fig. 4b and Supplementary Fig. S3b). Due to the fact that the binding receptor of C3b or iC3b fragments is CR1 or CR3 in macrophages^31,32^, we further compared the expression levels of CR1 and CR3 in the scRNA-seq and during *M. fortis* macrophage adhesion to schistosomula in vitro. Both results showed that the expression level of CR3 was higher than that of CR1 during the process of *M. fortis* macrophage adhesion to the schistosomula of *S. japonicum* (Supplementary Fig. S3c, d). These results suggest that C3 and its receptor CR3 may participate in macrophage adhesion-mediated schistosomula killing in *M. fortis*.

To further verify the role of C3 in *M. fortis* macrophages mediating effects on schistosomula, the co-culture system of macrophages and schistosomula was treated with different concentrations of normal *M. fortis* serum containing abundant C3. As shown in Fig. 4c, d, the numbers of macrophages that adhered to the schistosomula and the schistosomula killing rate were significantly increased after the addition of *M. fortis* serum in the culture medium (Fig. 4c, d) and strongly correlated with the concentration of serum (Fig. 4c, d). Cobra venom factor (CVF), a functional analog of C3b, forms a stable convertase that depletes serum C3 within hours^33^. To further explore the effects of the C3-CR3 pathway on cell adhesion and schistosomula killing, we used CVF to delete C3 in serum and anti-CD11b mAb to block CR3 function in macrophages in vitro and in vivo. Notably, our results showed that the blocking of the C3-CR3 pathway results in significant inhibition of macrophage adherence to schistosomula (Fig. 4e, f and Supplementary Videos S3–S5) and a reduction in the killing of schistosomula in vitro (Fig. 4g and Supplementary Videos S3–S5). Similarly, when C3 was exhausted by CVF treatment or CR3 function was blocked by anti-CD11b mAb in *M. fortis*, cell adhesion (Fig. 4h, i) and schistosome killing (Fig. 4j) were impaired as indicated by a higher worm load (Fig. 4j) and the increased size of schistosomes (Fig. 4k, l) in such animals. Thus, these data demonstrated that macrophage adhesion-mediated schistosome killing required complement C3 and its receptor CR3 in *M. fortis*.

### Trogocytosis participated in destroying schistosomes by *M. fortis* macrophages

**Fig. 5:**
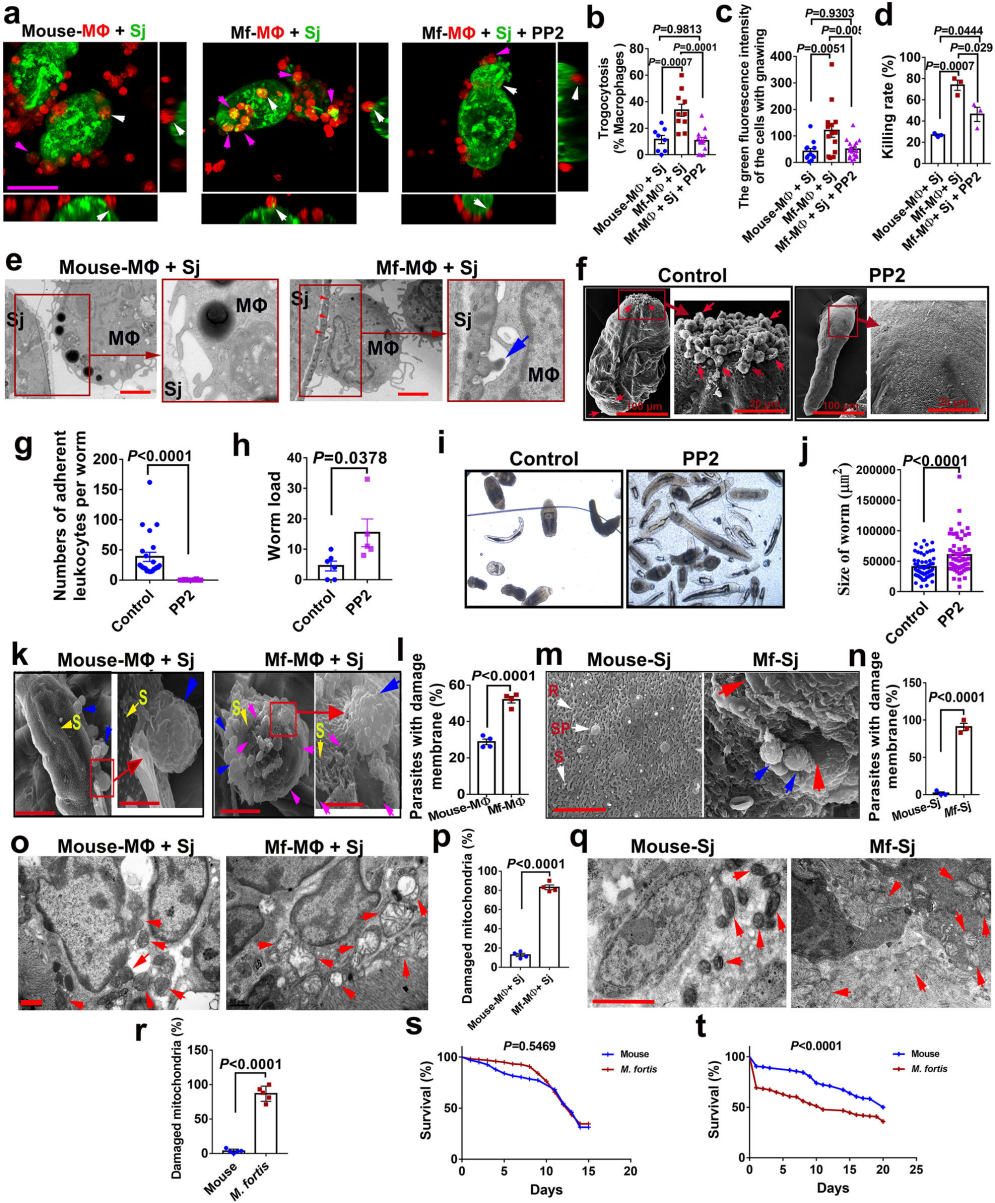
Trogocytosis participated in destroying schistosomes by *M. fortis* macrophages. **a** Representative images of macrophages from BALB/c mice and *M. fortis* showing trogocytic uptake of the schistosomula material. Macrophages from mice and *M. fortis* were labeled with Cell Tracker Deep Red (red), and the schistosomula was labeled with CDFA-SE (green). Macrophages and parasites were co-cultured at a ratio of 2000 macrophages:1 parasite in the presence of 5% normal mouse or *M. fortis* serum for 12 h, and the acquisition of labeled parasite material by macrophages (magenta arrows) was visualized by confocal fluorescent microscopy. The images were acquired by Z-stack spanning. The larger images in the middle are shown as a volume view, while the images on the right and below are shown as slices to visualize the inside of the macrophages, representing *YZ*-axis and *XZ*-axis, respectively. White arrows indicate the selected typical cell that adhered to the schistosomula. Scale bar, 50 μm. Mouse-MΦ: the macrophages from mouse; Sj: *S. japonicum*; Mf-MΦ: the macrophages from *M. fortis*; PP2: the inhibitor of trogocytosis, a potent and selective inhibitor of the src family of tyrosine kinases. **b** The percentage of macrophages that have taken up schistosomula material. **c** The number of “bites” of schistosomula material transferred to macrophages was quantified by measuring green fluorescence intensity in the macrophages. **d** The schistosomula killing rates cultured in the presence of 5% normal mouse or *M. fortis* serum for 12 h. Data shown are mean ± SEM and repeated thrice with similar results. **e** Conjugate formation and trogocytosis of macrophages (MΦ) and schistosomula (Sj) after co-culturing for 12 h were visualized by TEM. The mouse macrophages were loosely bound to schistosomula with no evidence of trogocytic invaginations, whereas the *M. fortis* macrophages were closely bound to schistosomula with trogocytic invaginations (red and blue arrows). Scale bar, 2 μm. **f**–**j** Analysis of leukocytes attached to the surface of *S. japonicum* (**f**, **g**), the worm load (**h**) and the development of *S. japonicum* (**i**, **j**) in vivo after PP2 or vehicle control (5% DMSO, 45% PEG300, 50% PBS) was injected into infected *M. fortis* at 13 days post-infection. The arrows indicate leukocyte adherence to schistosomes. *n* = 5–6. Data shown are mean ± SEM and repeated twice with similar results. **k** SEM images of the trogocytic outcomes on the surface of schistosomula by macrophages (blue arrows) isolated from mouse (Mouse-MΦ) and *M. fortis* (Mf-MΦ) after incubation for 16 h in the presence of 5% mice or *M. fortis* serum. S, spine. Yellow arrows show the remaining spines in the epidermis of the schistosomula; magenta arrows show the exposed muscle layer through the bitten-off epidermis of the schistosomula. The whole worm was photographed at 5000× (scale bars, 20 μm); local parts of the worm were photographed at 20,000× (scale bars, 5 μm). **l** Statistical analysis of the percentage of schistosomula with damaged membrane in vitro from **k**. **m** SEM images of the trogocytic outcomes of *S. japonicum* by leukocytes (blue arrows) in the mouse and *M. fortis* (Mf) 21 days post-infection. Sj *S. japonicum*, R ridge, S spine, SP sensory papillae. Red arrows show the exposed muscle layer with the exfoliated epidermis. Scale bars, 10 μm. **n** Statistical analysis of the percentage of *S. japonicum* with damaged membrane in vivo from **m**. **o** Ultrastructural analysis of mitochondria in schistosomula after incubation with macrophages (either mice or *M. fortis*) for 12 h in the presence of 5% mice or *M. fortis* serum. Arrows indicate mitochondria. Scale bars, 0.5 μm. **p** Statistical analysis of the percentage of damaged mitochondria of schistosomula in vitro from o. **q** Ultrastructural observation of the mitochondria of schistosomula collected from mice and *M. fortis* infected with *S. japonicum* post 13 days. Arrows indicate mitochondria. Scale bars, 2 μm. **r** Statistical analysis of the percentage of damaged mitochondria of *S. japonicum* in vivo from **q**. **s** Percentage survival of *S. japonicum* collected from infected BALB/c mice (blue line) and *M. fortis* (red line) on day 7 post-infection in vitro culture. No cell adhesion and no epidermal damage were found on the surface of worms from both hosts at this stage. **t** Percentage survival of *S. japonicum* collected from infected BALB/c mice (blue line) and *M. fortis* (red line) on day 14 post-infection in vitro culture. No cell adhesion and no epidermal damage were found on the surface of worms from BALB/c mice at this stage. In contrast, the worms from *M. fortis* have cell adhesion and epidermal damage at this stage. All data are expressed as the mean ± SEM of 4**–**6 animals per group. Similar results were obtained in at least three repeat experiments.

With scRNA-seq analysis, we found that the adherent cells exhibited higher activity in the pathway of endocytosis and phagocytic process (Fig. 2c). However, because the size of the schistosome is much larger than that of macrophages, the macrophages cannot endocytose or phagocytose the worm. To explore the mechanism of macrophage adhesion-mediated schistosomula killing, the macrophages of BALB/c mice and *M. fortis* were labeled with Cell Tracker Deep Red (red) and the schistosomula membranes were labeled with green fluorescence, and the interaction between macrophages and schistosomula in the co-culture system was observed by laser scanning confocal microscopy. Surprisingly, compared with the lack of detection or limited detection of schistosomula material in the mouse macrophages (Fig. 5a and Supplementary Video S6), we observed that the attachment of *M. fortis* macrophages led to the uptake of a large amount of schistosomula material in these macrophages (Fig. 5a and Supplementary Video S7) in a way that was reminiscent of trogocytosis. Trogocytosis is a process by which a cell takes a “bite” of a neighboring cell, a process also referred to as “nibbling”. Interestingly, our subsequent ultrastructural analysis results showed that *M. fortis* macrophages were closely bound to schistosomula and trogocytic invaginations were observed (Fig. 5e). This is a classic observation when cells take “bites” of their targets^34^. In contrast, the mouse macrophages made loose contact with the schistosomula with no obvious trogocytic invaginations (Fig. 5e). The statistical analysis showed that the numbers of trogocytosis-positive cells (Fig. 5b) and the intensity of nibbling (intracellular green fluorescence intensity) (Fig. 5c) of *M. fortis* macrophages were significantly higher than those of mouse macrophages. Furthermore, this behavior of *M. fortis* macrophages was able to be suppressed by PP2 (a selective inhibitor of the Src family of tyrosine kinases) (Fig. 5a–c and Supplementary Video S8) and cytochalasin D (an inhibitor of actin filament formation)^35^ (Supplementary Fig. S4a–d), which were used as the inhibitors of trogocytosis^36,37^. As a result, treatment with PP2 and cytochalasin D resulted in a significant reduction in schistosomula killing by *M. fortis* macrophages (Fig. 5d and Supplementary Fig. S4e). Similarly, when PP2 was administered to *M. fortis*, cell adhesion (Fig. 5f, g) and schistosome killing (Fig. 5h) were inhibited, as demonstrated by the increased parasite load (Fig. 5h) and the worm size (Fig. 5i, j) in the PP2-treated animals. The results suggest that intimate contact-dependent trogocytosis participates in the killing of schistosomes by *M. fortis* macrophages.

Furthermore, we monitored the destruction of the schistosome epidermis with SEM to investigate whether macrophage trogocytosis was responsible for the death of schistosomula. In the co-culture system of macrophages and schistosomula in vitro, SEM showed that the percentage of the schistosomula with damaged membrane co-cultured with *M. fortis* macrophages was significantly higher than that of schistosomula co-cultured with mouse macrophages (Fig. 5l). As shown in Fig. 5k, l, 52% ± 1.8 of the teguments belonging to the schistosomula in the *M. fortis* macrophage group were broken and stripped in patch form (magenta arrows). The damage was consistent with being gnawed by macrophages (blue arrows), and the spines on the remaining unstripped epidermis were scattered or had disappeared. Similarly, the damaged, stripped tegument and even the exposed muscle layer of *S. japonicum* was also observed in vivo in *M. fortis*, while this phenomenon was rarely or never found on the schistosome epidermis in mice (Fig. 5m, n). These results were also confirmed for the related schistosome species *S. mansoni* (Supplementary Fig. S4f, g). In addition, transmission electron microscopy (TEM) ultrastructural results revealed that most of the mitochondria of the schistosomula co-cultured with *M. fortis* macrophages in vitro (Fig. 5o, p) and collected from *M. fortis* in vivo (Fig. 5q, r) showed swelling, distortion, loss of intact internal membranes and disruption of mitochondrial cristae with vacuolization. In contrast, the schistosomula co-cultured with mouse macrophages presented intact mitochondria with well-defined outer membranes and clear structures of cristae (Fig. 5o), and the same observations were made when collected from mice in vivo (Fig. 5q). Both observations are consistent with the killing occurring by trogocytosis. Furthermore, when 7-day and 14-day *S. japonicum* were taken from infected mice and *M. fortis* and cultured in vitro, the results showed that there was no significant difference in the survival rate of 7-day schistosomula from the two hosts (both of which had no cell adhesion and epidermal damage) (*P* > 0.05) (Fig. 5s), while the survival rate of 14-day schistosomula from *M. fortis* (with cell adhesion and epidermal damage) was significantly lower than that of 14-day schistosomula from mice (no cell adhesion and no epidermal damage) (*P* *<* 0.0001) (Fig. 5t). Therefore, again, these results further demonstrated that trogocytosis is involved in the killing of schistosomes in *M. fortis*.

### Ca^2+^/NFAT signaling regulates macrophage adhesion-mediated destruction of schistosomes in *M. fortis*

**Fig. 6:**
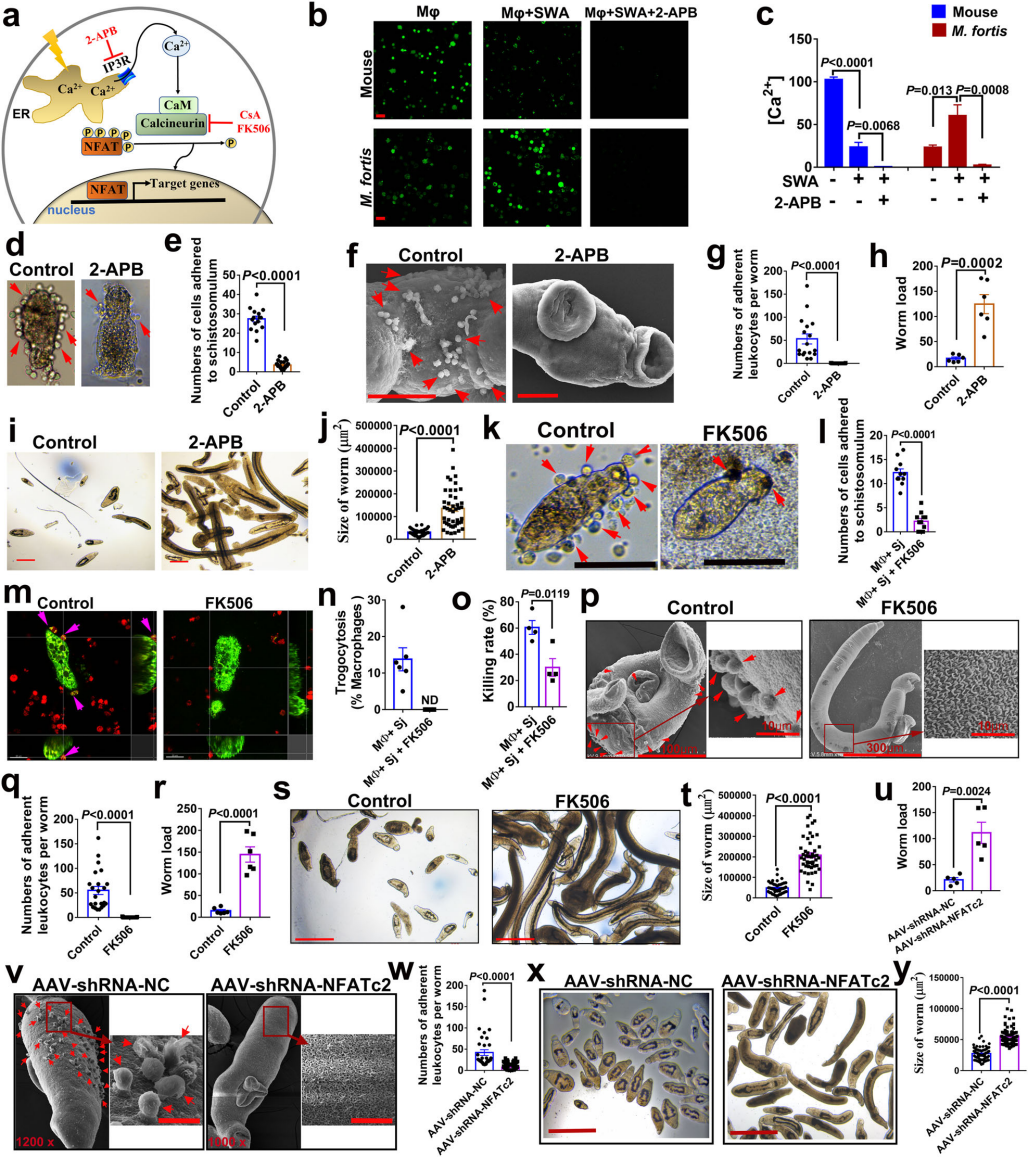
Cell adhesion-mediated destruction of schistosomes was inhibited after blocking NFAT activation in vitro and in vivo. **a** A diagram of the Ca^2+^/NFAT signaling pathway and inhibitors involved in NFAT activation. The IP3 receptor can be antagonized by 2-aminoethoxydiphenyl borate (2-APB) to inhibit the release of Ca^2+^ from the endoplasmic reticulum. The activity of calcineurin and NFATc function can be specifically blocked by the immunosuppressive drugs cyclosporine A and FK506. **b**, **c** Detection of cytosolic Ca^2+^ levels. Macrophages from mouse or *M. fortis* were stimulated with schistosome worm antigens (SWA, 10 μg/mL) for 6 h and then loaded with Fluo-4-AM to determine the Ca^2+^ levels and measured by confocal microscopy. Representative confocal images (**b**). Scale bars, 20 μm. Quantification of the concentrations of Ca^2+^ by Fluo-4 fluorescence (**c**). *n* = 3 mice or *M. fortis*. Data represent mean ± SEM and repeated thrice with similar results. **d**, **e** Analyzing the effects on the numbers of cells adhering to schistosomula after inhibiting the release of Ca^2+^ from the endoplasmic reticulum with 2-APB in vitro. Equal volume of PBS as vehicle control. Representative micrographs of macrophages adherence to schistosomula (**d**). The arrows indicate macrophage adherence to schistosomula (**d**). And then quantitative analysis of the numbers of cells attached to schistosomula (**e**). *n* = 3 *M. fortis*. Data represent mean ± SEM and repeated thrice with similar results. **f**–**j** Analysis of leukocytes attached to the surface of *S. japonicum* (**f**, **g**), the worm load (**h**) and development of *S. japonicum* (**i**, **j**) in vivo after 2-APB or vehicle control (PBS) was injected into infected *M. fortis* at 13 days post-infection. The arrows indicate leukocyte adherence to schistosomes. *n* = 6 *M. fortis* per group. Data shown are mean ± SEM and representative of at least three independent experiments. **k**–**p** Analyzing the effects on the numbers of cells adhering to schistosomula (**k**, **l**; scale bars, 100 μm), trogocytosis (**m**, **n**) and the schistosomula killing rate (**o**) after blocking NFAT activation with FK506 in vitro. Equal volume of DMSO as vehicle control. The red arrows indicate macrophage adherence to schistosomula. Magenta arrows indicate the macrophages with positive trogocytosis. ND not detected. *n* = 3 *M. fortis*. Data represent mean ± SEM and repeated twice with similar results. **p**–**t** After blocking NFAT activation with FK506 in *M. fortis*, the leukocyte adhesion on the surface of *S. japonicum* (**p**, **q**), the worm load (**r**), and development of *S. japonicum* (**s**, **t**, scale bars, 500 μm) were compared. The arrows indicate leukocyte adherence to schistosomes. Equal volumes of corn oil were used as vehicle control. All data are expressed as the mean ± SEM of 6 animals per group. Data are representative of three independent experiments. **v**–**y** AAV-shRNA-NFATc2 inhibited leukocyte adherence to schistosomes (**v**, **w**, scale bars, 10 μm), increased worm load (**u**) and worm sizes (**x**, **y**, scale bars, 500 μm) in *M. fortis*. All data are expressed as the mean ± SEM of 5 animals per group and representative of two independent experiments.

Since the regulatory network analysis of scRNA-seq results showed that the NFAT family (NFATc2 and NFATc3) were in the top list of activated TFs (Fig. 2d), we further investigated whether NFAT regulates the process of cell adhesion-mediated schistosome killing in *M. fortis*. NFAT plays an important role in immune response, and the activation of NFAT signaling requires the increase of intracellular Ca^2 +^ and calcineurin, which dephosphorylates the NFATc proteins (c1 to c4), causing NFATc to translocate into the nucleus and inducing target gene expression (Fig. 6a)^38^. As shown in Supplementary Fig. S5a, b, the expression of NFATc2 and c3 in the liver of *M. fortis* were significantly increased at day 13 post-infection, at which time the amount of cell adherence was the greatest. However, the increased levels of NFATc2 and c3 post-infection were not found in mice at this time, which indicates that NFAT may play a role in resistance to *Schistosoma* infection in *M. fortis*. Interestingly, we found that the cytosolic Ca^2+^ levels in *M. fortis* macrophages were significantly increased after stimulation with schistosome worm antigens (SWA) (Fig. 6b, c), while the increase in Ca^2+^ levels was not found in mouse macrophages (Fig. 6b, c). In addition, the increase of cytosolic Ca^2+^ level in *M. fortis* macrophages was inhibited by 2-aminoethoxydiphenyl borate (2-APB, store-operated Ca^2+^ channel blocker, inhibiting the release of Ca^2+^ from the endoplasmic reticulum) (Fig. 6b, c). Notably, when the cytosolic Ca^2+^ level in *M. fortis* macrophages was inhibited by 2-APB, a significant reduction in the number of cells adhering to the schistosomula was found (Fig. 6d, e and Supplementary Video S9). In addition, the effects of NFAT signaling activated by elevated Ca^2+^ levels on cell adhesion and the killing of schistosomes were further verified in vivo. The results showed that the cell adhesion (Fig. 6f, g) and schistosome killing (Fig. 6h) were impaired, as indicated by higher worm loads (Fig. 6h) and the larger sizes of schistosomes (Fig. 6i, j) in *M. fortis* treated with 2-APB, compared with the control group.

**Fig. 7:**
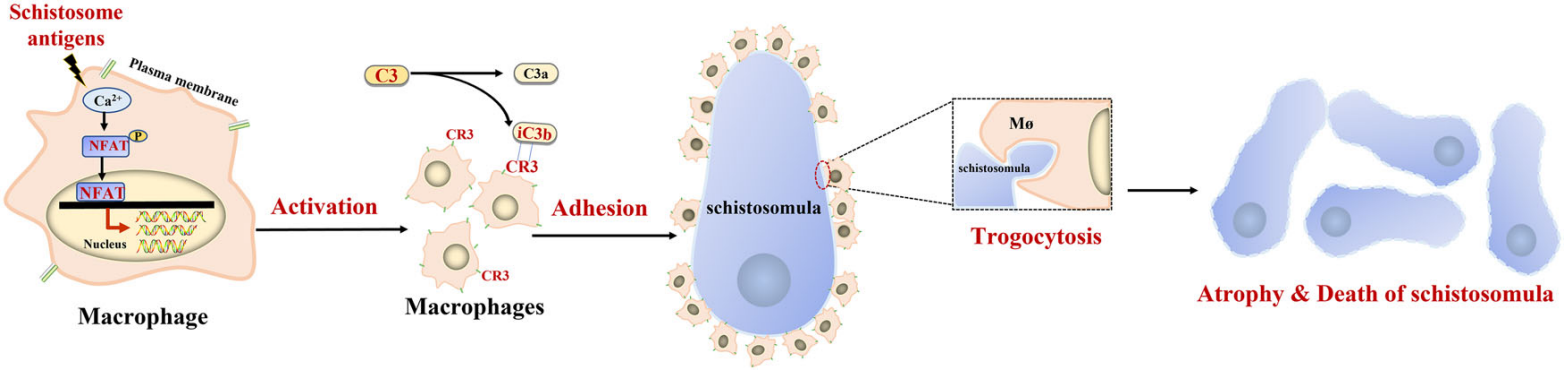
Schematic model for the process of macrophage-mediated trogocytosis in destroying human schistosomes. Stimulation of *Schistosoma* antigens leads to an increase in intracellular Ca^2+^ concentration in *M. fortis* macrophages, further inducing the activation of NFAT. The activation of NFAT can induce the transcriptional expression of multiple genes, causing macrophage adhesion to the surface of schistosomula through C3–CR3 interaction. Subsequently, the macrophages destroy the parasites through trogocytosis, ultimately leading to impaired growth, atrophy, and even death of schistosomula.

It is reported that the activation of NFAT can be efficiently inhibited by the immunosuppressive drugs cyclosporine A and FK506 (Fig. 6a)^39^. To further investigate the role of NFAT in regulating the process of adhesion-mediated killing of *Schistosoma* by immune cells of *M. fortis*, we blocked NFAT function with FK506 in vitro and in vivo. As demonstrated in Fig. 6k–n, FK506 effectively reduced the number of cells that adhered to the schistosomulum (Fig. 6k, l) and reduced macrophage trogocytosis (Fig. 6m, n), resulting in a significant reduction in schistosomulum killing (Fig. 6o). In vivo, blocking of the NFAT signal with FK506 efficiently downregulated NFAT expression in the liver of *S. japonicum*-infected *M. fortis* (Supplementary Fig. S5c, d). Notably, compared with the control group, the blocking function of NFAT in *M. fortis* led to impaired leukocyte adherence to schistosomes (Fig. 6p, q) and increases in the number of larger sizes of parasites (Fig. 6r–t). We further used an adeno-associated virus serotype 8 (AAV-shRNA-NFATc2) to knock down Nfatc2 in *M. fortis*, and we obtained similar results with FK506 to inhibit NFAT pathway (Fig. 6u–y). Together, these findings indicate that the Ca^2+^/NFAT signaling pathway is involved in the cell adhesion-mediated macrophage trogocytosis mechanism for killing *S. japonicum* (Fig. 7).

## Discussion

Host specificity is a very complicated and fascinating interaction between animal hosts and pathogen infections. This phenomenon is a reflection of the biodiversity and evolution of the relationship between the parasite and its host. For instance, human resistance against animal trypanosomes (e.g., *Trypanosoma brucei*) infection relies on the effect of serum complexes where apolipoprotein A-1 (apoA1) associates with two primate-specific proteins, apolipoprotein L-1 (apoL1) and haptoglobin-related protein (Hpr)^40,41^, while the interaction of the Lewis rat and iNOS knockout SD rat with *Toxoplasma* infections have been suggested to link with pyroptosis^42,43^. Although many studies have been done on parasitic protozoa, there are still a lot of enigmas surrounding the remarkable differences in the susceptibility among hosts toward helminth infections. The resistance of *M. fortis* against schistosome infection is a typical example^44^. In the last few decades, although a large number of studies have been undertaken to explore the mechanism behind this efficient resistance in *M. fortis*, the underlying mechanisms remain poorly understood. In this study, using SEM analysis, we found that numerous immune cells attached to the surface of schistosomula in *M. fortis* infected with either *S. japonicum* or *S. mansoni*, while this phenomenon was absent in other hosts we examined. To the best of our knowledge, the phenomenon has not yet been reported in vivo by previous investigators. Based on the results of scRNA-seq analysis, macrophages were identified as an important group of cells that may exert these schistosomulicidal effects. Interestingly, early studies indicated that the activated human and mouse macrophages could mediate cytotoxicity to the schistosomula of *S. mansoni* via a contact-dependent manner in vitro^45–47^. Notably, our results revealed that the ability of macrophages isolated from the normal *M. fortis* to adhere and destroy the schistosomula was significantly higher than those from the normal mouse under the same conditions of in vitro cultivation, which was consistent with the phenomenon we found in these animals. The role of macrophage-mediated attachment and destruction of schistosomula in vivo was further demonstrated by the depletion of macrophages with clodronate liposomes in *M. fortis*.

Previous investigators speculated that the attachment of macrophages to the schistosomula could promote local high concentrations of secreted molecules to damage the parasite, but results showed that the conventional macrophage cytotoxic secretory products, such as TNF, proteases and products of oxygen metabolism, had no obvious cytotoxic effects on schistosomula in in vitro cultivation^47^. Therefore, although the in vitro effects of mammalian macrophage-mediated cytotoxicity on the schistosomula, mediated by cell contact, have been known for many years, the detailed mechanisms were yet to be elucidated. Generally speaking, in addition to the production of antibacterial mediators, phagocytosis is a canonical pathway used by macrophages for immune defense^25^. However, since *Schistosoma* species are relatively big multicellular organisms, it is unlikely that macrophages could engulf such a target. Intriguingly, our direct evidence demonstrated that macrophages performed trogocytosis, a process by which one cell contacts and quickly “bites” the membrane of a neighboring cell, resulting in the death of the schistosomula. To our knowledge, this is an unprecedented way for immune cells to kill helminths in mammals and is the first time that it has been reported. In fact, previous studies on trogocytosis have been mostly described as a mechanism of transfer of membrane proteins between immune cells. It has been implicated as being widely involved in antigen presentation, information transmission and modulation of immune cell functions^48^. Trogocytosis-mediated cell death was first described in the unicellular parasite *Entamoeba histolytica*, where the parasite kills living human cells by ingesting pieces of cell^49^. Recently, neutrophils and macrophages have also been identified to perform trogocytosis-mediated killing of the unicellular parasite *Trichomonas vaginalis*^50^ and antibody-opsonized tumor cells^51^ in vitro. In addition, trogocytosis-mediated antigen loss on target cells has been shown to decrease the efficacies of chimeric antigen receptor (CAR) T cell therapy^52,53^ and antibody-based therapies^54,55^. Thus, our study reporting the novel finding of trogocytosis-mediated destruction of the large multicellular parasite *Schistosoma* adds another dimension to this interesting mechanism. All these paths of evidence indicate that trogocytosis may be a neglected but common mechanism used by mammalian hosts against parasites from protozoa through to helminth infections. As a matter of fact, a similar killing mode was also found in vaginal neutrophils, which destroyed sperm in vitro^56^. Indeed, current reports of mammalian immune cells performing trogocytosis-mediated killing are mainly focused on neutrophils^34,50^. Thus, whether *M. fortis* neutrophils can operate through a similar mechanism to destroy schistosomula remains to be studied in the future.

Schistosomula death is most likely due to the accumulation of membrane damage. Using in vitro and in vivo analysis with SEM, we clearly observed that the tegument (membrane) of schistosomula, of both *S. japonicum* and *S. mansoni*, was damaged by *M. fortis* macrophages by being stripped in patch form, most likely being the result of the “nibbling” associated with trogocytosis. It is well known that the intact tegument is necessary for the survival of schistosomes and for their resistance against host immune attack^57^. Therefore, any damage to the tegument could lead to the destruction of its barrier function. This phenomenon is similar to that of *E. histolytica* killing host cells by trogocytosis resulting in intracellular calcium elevation and eventual cell death^49^. The findings from our current work in vitro and in vivo with TEM analysis also supported this view and showed that most of the mitochondria of schistosomula, subjected to macrophage attack by *M. fortis*, were swollen and distorted. Furthermore, this idea was further supported by the results of survival differences between 7-day and 14-day-old schistosomula collected from mice and *M. fortis* in vitro cultivation, which indicated that the survival rate of worms with cell adhesion was significantly reduced. On the other hand, the destruction of the tegument might also lead to the exposure of schistosome antigens, which could trigger the host’s immune system to recruit other inflammatory cells, such as neutrophils, eosinophils and macrophages, to attack the surface of worm.

In addition, we further demonstrated that the *M. fortis* macrophage-mediated trogocytosis on schistosomula, via adherence, required complement-mediated opsonization. Furthermore, these *M. fortis* macrophages could efficiently recognize C3b/iC3b-tagged schistosomula by the receptors CR3 on their surface, promoting trogocytosis and destroying the parasites. Although both CR1 and CR3 are complement receptors involved in the recognition of C3b/iC3b fragments, they have different functions. CR1 mainly facilitates the clearance of complement-opsonized particles, whereas CR3 (expressed at higher levels in *M. fortis* macrophages than CR1 during the process of macrophages adhesion to schistosomula) is primarily involved in phagocytosis, leukocyte adhesion, and synapse formation^31,32^. Due to the contact-dependent nature of trogocytosis, cell adhesion and synapse formation is a prerequisite for trogocytosis^48,49^. Therefore, we believe that the C3-CR3 recognition pattern plays a dominant role in the trogocytosis of *M. fortis* macrophages. This is consistent with earlier studies in leukocytes from rats and humans which reported that eosinophils required to activate complement or exploit CR3 for adhesion and subsequent cytotoxicity against schistosomula in vitro^58,59^, although trogocytosis in these studies was not considered as a mechanism in killing activity. Similarly, it is reported that CR3-mediated adhesion of neutrophils and eosinophils to cancer cells and led to tumor cell killing^34,60^. In contrast, mouse macrophages exhibited a weaker ability to recognize iC3b-opsonized schistosomula. These results indicate that there is an intrinsic difference in macrophage behavior between these two species (permissive host vs non-permissive host) in response to schistosome infection. It has to be mentioned that, in addition to the opsonization of complement to mediate cell adhesion, C3 is an essential component in the formation of the membrane attack complex (MAC) mediating membrane-penetrating lesions that lead to cell death^61^. In *M. fortis*, previous studies have shown that the Complement in the serum of *M. fortis* can directly exert cytotoxicity to schistosomula in vitro^62^. These results do not indicate that Complement plays an absolutely independent role in the anti-schistosomes in *M. fortis*. For example, it is well known that guinea pig serum, exhibiting the highest Complement activity among many experimental animals^63^, can quickly kill all the schistosomula within 2 h, and the killing efficiency was much higher than that of *M. fortis* serum in vitro (our results). However, the existing studies have shown that the development of schistosomes in guinea pigs was much better than those found in *M. fortis*, and some schistosomes were able to mature and lay eggs in the liver^64,65^. In contrast, all evidence indicated that schistosomes in *M. fortis* could only maintain the schistosomula status. These results suggest that the factors in vivo are much more complex than those in vitro, and the results performed in vitro may sometimes be limited when interpreting the in vivo situation. Therefore, we consider that the powerful anti-schistosome properties of *M. fortis* could be the result of the interaction between the macrophages and extrinsic factors (C3), and is the result of long-term interaction (evolution) between schistosomes and host.

An important question is why, despite stimulation with the same schistosome antigens, can *M. fortis* (the non-permissive host) macrophages initiate trogocytosis to attack the worm but mouse (the permissive host) macrophages cannot? Our results demonstrated the Ca^2+^/NFAT signaling pathway is involved in the cell adhesion-mediated macrophage trogocytosis mechanism for destroying *S. japonicum*. It is very interesting that the cytosolic Ca^2+^ levels in *M. fortis* macrophages were significantly increased after stimulation with schistosome worm antigens, while mouse macrophages were not, which indicates mouse macrophages may not be activated. Previous studies demonstrated that murine and human macrophages exert effective cytotoxicity against schistosomula of *S. mansoni* in vitro only by activated macrophages stimulated by agents (such as Bacille Calmette-Guerin, *Mycobacterium bovis*, IFN-γ and LPS)^45–47^. In addition, an inherent concept was the idea that, by analogy to phagocytosis, it was the activating receptors on immune effector cells that promoted trogocytosis^55^. Moreover, the activation of NFAT induced by intracellular Ca^2 +^ has been shown to modulate various biological functions of immune cells^38^. Earlier studies indicated that NFAT could regulate the recruitment of effector cells to inflammatory sites to participate in host defense by regulating the transcription of chemokines and inflammatory factors^66–68^. In addition, many studies have indicated that NFAT has a well-established role in promoting cell adhesion and migration^69–71^, which is consistent with a mechanism in which adhesion-dependent trogocytosis is regulated by NFAT. Interestingly, Pontrelli and colleagues reported that cyclosporine A (another inhibitor of NFAT signaling) could inhibit cytoskeletal reorganization^71^, which is a key requirement for amebic trogocytosis^49^.

In summary, by observing the adhesion properties of *M. fortis* macrophages toward schistosomula, both in vivo and in vitro, we have discovered a novel way of destroying worms by mammalian macrophages, a process referred to as trogocytosis. C3 and its receptor CR3 are required for this contact-dependent destruction, and the process can be governed by Ca^2+^/NFAT signaling. This work not only elucidates the anti-schistosome mechanism in *M. fortis* but also offers insights into a better understanding of host–parasite interactions, host specificity and the potential generation of novel strategies for schistosomiasis control. As a matter of fact, macrophage-mediated destruction of the schistosomula bears many similarities to other important immunological destructive processes, such as tumor and graft cytotoxicity in other species, including humans. In addition to providing knowledge that could inform novel strategies for understanding the diversity of the host specificity mechanisms and the evolution of the relationship between host and parasite, we speculate that these findings may even also offer a new perspective for therapies in cancer and organ transplantation.

## Materials and methods

### Parasites

Cercariae of *S. japonicum* (Chinese mainland strain) were obtained from infected *Oncomelania hupensis* snails purchased from the Institute of Parasitic Diseases of China CDC in Shanghai. The *S. mansoni* strain was provided by the Jiangsu Institute of Parasitic Diseases, Wuxi, Jiangsu, China. To obtain SWA, *S. japonicum* was collected by perfusion of the hepatic portal system on day 14 post-infection from mice. The worms were homogenized in PBS after being washed three times and subjected to five cycles of rapid freezing and thawing. The suspension was centrifuged at 12,000× *g* for 30 min at 4 °C and the supernatants were collected, and the protein concentrations were determined using a BCA kit.

### Animals

Six to eight-week-old male Bagg albino (BALB/c) mice were purchased from the Medical Experimental Animal Center of Guangdong Province, Guangzhou, China. Six to eight-week-old male *M. fortis* were provided by the Animal Health Center, Xiangya School of Medicine, Central South University, Hunan, China. All animal work was performed according to the recommendations of the Laboratory of Animal Welfare and Ethics Committee (LAWEC) of China and approved by the LAWEC of Sun Yat-Sen University under license number SYSU-IACUC-2020-B0113 and Central South University under license number 2017sydw0237. All animals were euthanized by cervical dislocation under anesthesia.

### Animal infection and sample collections

Based on the rodent species’ susceptibility to *Schistosoma*, each mouse was percutaneously infected with 40 cercariae and *M. fortis* were exposed percutaneously to 200 cercariae, except in experiments designed to collect worms, when a dose of 1000 cercariae was used. Parasites were harvested by perfusion from the portal system. The collected worms were counted and photographed under a stereoscopic microscope (M205FA, Leica, Germany). The size of worms was measured from micrographs using the Image-Pro Plus 6.0 software. The livers of the animals were removed for pathological and molecular transcriptional analysis.

### SEM analysis

SEM was performed as previously described^72^. In brief, the schistosome samples were fixed in 0.2 M PBS, pH 7.4, containing 2.5% glutaraldehyde for over 24 h at 4 °C. After dehydration in an ethanol gradient from 50% to 100% (v/v), the samples were soaked sequentially with acetone and isoamyl acetate. Following critical point-drying, samples were pasted in the sample stage and then coated with gold in an ion coater (Hitachi E-102, Japan). Parasites were observed and photographed using a scanning electron microscope (Hitachi S-2500, Japan). The number of leukocytes adhering to the surface of the schistosome was counted under the scanning electron microscope.

### Single cell preparation and sequencing

After *M. fortis* were infected with 1000 *S. japonicum* cercariae, parasites were harvested by perfusion at 13 days post-infection. The collected worms were washed five times in PBS plus 0.04% bovine serum albumin (BSA) (Sigma). Each wash was performed by sedimentation and resuspension in 1 mL PBS plus 0.04% BSA. The adhered leukocytes on the surface of the schistosomula were separated with trypsin. Briefly, the worms were resuspended in 0.25% trypsin-EDTA (Gibco 25200056) for 1 min at 37 °C, then kept at room temperature, and the state of digestion was dynamically observed under a microscope (occasionally bouncing the culture dish with fingers to loosen cells). The enzymatic digestion was stopped with 10 times the volume of PBS (containing 0.04% BSA) when the adhered leukocytes on the surface of the schistosomula detached from the parasites. The suspension was filtered through a 40 µm cell strainer and centrifuged at 500 × *g* for 5 min. The cell pellet was resuspended in PBS (containing 0.04% BSA). Cell viability was determined using Trypan blue staining. Then the dissociated single cells were picked promptly by pipette under a microscope and immediately transferred into a prechilled 96-well plate containing 2 μL lysis buffer. The cell lysis buffer contained 2U RNase inhibitor (Takara, China), 0.0475% Triton X-100 (Sigma-Aldrich, USA), 2.5 μM dNTP mixture (Thermo, USA), and 0.75 μM tailed oligo-dT oligonucleotides. Single-cell libraries were prepared according to the Smart-seq2 protocol as described previously^22^. Each sequencing library was generated with a unique sample index. The libraries were sequenced on the NovaSeq 6000 platform at Annoroad Gene Technology (Beijing, China) with 150-bp paired-end reads.

### scRNA-seq analysis

We first removed the contamination of *S. japonicum* mRNA before analysis. Briefly, 56,323 nucleic acid sequences of *S. japonicum* were downloaded from the NCBI database, including genomic DNA sequence and mRNA sequence, and the *S. japonicum* index, which was required for bowtie2 sequence alignment, was constructed. For the original sequencing data of *M. fortis*, Trim Galore was used to remove the joint sequence, and Fastqc was used for quality control. Then, bowtie2 was used to align the sequencing data of *M. fortis* with the *S. japonicum* index, which obtained the reads that aligned concordantly with the *S. japonicum* index and those reads that were unmatched. The latter unmatched reads, with the *S. japonicum* index, were considered as the clean reads from *M. fortis* for downstream analysis.

Clean reads were then aligned to the mouse genomes (GRCm39) and *M. fortis* transcriptomes (obtained by using StringTie to assemble traditional transcriptome sequencing data and single-cell transcriptome data, and then annotation by blast) using STARsolo aligner with the flags “--outFilterScoreMinOverLread 0.1 --outFilterMatchNminOverLread 0.1”, respectively. To exclude low-quality cells in both single-cell experiments, we filtered out cells that detected fewer than 200 genes per cell.

### Dimension reduction and clustering

Seurat (V4) was used to perform processing of the scRNA-seq data. The filtered gene-cell matrix was then normalized and scaled. Uniform manifold approximation and projection (UMAP) and clustering were performed based on 1500 variably expressed genes with the optimal number of principal components (first 4 principal components from principal component analysis).

### Pathway enrichment and regulatory network analysis

Pathway enrichment analysis of the top 200 highly expressed genes was performed using Metascape^73^ (https://metascape.org). Single Cell rEgulatory Network Inference and Clustering (SCENIC) was used to build a regulatory network and calculate the activity of regulon (the gene set of TF and its target genes) based on aligned results of mouse^26^. To screen key TFs that regular macrophage adhesion-mediated schistosomula killing, the score was first transformed to a binary of 0 and 1 to represent the activation status of regulons. The regulons with any other regulon correlated (absolute value of Pearson correlation coefficient > 0.30) and active in at least 1% of cells were reserved.

### Preparation of schistosomula

Schistosomula were obtained from cercariae by a mechanical method in vitro, as described previously, with minor modifications^58^. Briefly, the fresh cercariae that were shed from snails were collected in cold deionized water containing penicillin and streptomycin and centrifuged at 100× *g* for 3 min. 1 mL of deionized water was added to the pellet, and the suspension was repeatedly sucked and pumped out with a 1 mL syringe until the tails of most cercariae were ruptured. The cercarial bodies were separated by sedimentation in PBS and washed three times to ensure sterility before being added to the cytotoxicity assay. The schistosomula were used on the day of preparation.

### Macrophage preparations

Peritoneal macrophages were isolated as previously described^72^. Briefly, mice and *Microtus fortis* were intraperitoneally injected with cold PBS containing penicillin and streptomycin to wash the peritoneal cavity after the animals were sacrificed. The peritoneal exudate cells were recovered and centrifuged at 250× *g* for 10 min. After being washed twice and resuspended in RPMI-1640 medium (GIBCO, USA), the cell suspensions were counted and seeded into 96-well culture plates (5 × 10^4^ cells/well). Each well was then washed three times with PBS to remove nonadherent cells following incubation for 2 h at 37 °C. Then, the cells were cultured in fresh RPMI-1640 medium supplementary with 10% heat-inactivated fetal bovine serum (FBS) (GIBCO, USA) and penicillin (100 U/mL) and streptomycin (100 mg/mL).

### In vitro cell adherence and schistosomulum killing assays

The assay was performed basically as described previously^45,74^ with minor modifications. Briefly, macrophages and schistosomula were co-cultured at an effector cell: target ratio of (1–2) × 10^3^: 1 in RPMI-1640 supplemented with 10% heat-inactivated FBS (except for the initial comparison of the adherence and schistosomulum killing of *M. fortis* and mouse macrophages, a non-heat-inactivated FBS was used), penicillin (100 U/mL) and streptomycin (100 mg/mL). In some experiments, as stated in the figure legends, 5% serum collected from normal mouse or *M. fortis*, 5% C3-inactivated *M. fortis* serum by CVF, CD11b mAb (20 µg/mL; BioXcell, USA), PP2 (20 µM; Sigma-Aldrich, USA), cytochalasin D (10 µM; Sigma-Aldrich, USA), 2-Aminoethyl diphenylborinate (2-APB, 10 µM; Sigma-Aldrich, USA) and FK506 (10 ng/mL; Sigma-Aldrich, USA) were additionally added to the cultures, respectively. In no case did these reagents demonstrate any direct toxicity toward the schistosomulum at the concentrations used. After 12–24 h of incubation at 37 °C in 5% CO_2_, cell adherence and schistosomula killing were evaluated. Schistosomula killing was determined microscopically by the criteria of loss of motility and internal granularity^46,74^. The numbers of adherent cells on the schistosomulum were counted under a microscope. For dynamic detection of the cell adhesion and schistosomulum vitality after incubation for 0–12 h, live cell imaging was started within 10 min after initiation of the experiment and was performed at various indicated times and intervals in an Operetta CLS™ high-content analysis system (Perkin Elmer, USA). Images were acquired using the 20× objective and analyzed using the Harmony software on the *Operetta CLS* (*Perkin Elmer*).

### Reproductive organ examination of schistosomes

Examination of schistosome reproductive organs was conducted following a protocol previously described^72^. Briefly, the schistosomes were fixed in 4% paraformaldehyde (PFA). Following washing with PBS, the worms were stained with 2.5% hydrochloric carmine red (Merck, USA) for 1 h and then destained in 70% acidic ethanol. After being dehydrated in an ethanol gradient, the parasites were clarified in methyl salicylate and preserved as whole mounts on glass slides. Samples were observed in a light microscope (Leica, Germany) and a laser scanning confocal microscope (LSM780, Zeiss, Germany) with a 488 nm laser and a 470 nm long-pass filter under reflection mode.

### Histopathology

Liver tissues were fixed in 4% PFA and then embedded in paraffin. Sections were obtained using an ultra-microtome and collected on glass slides. Following dewaxing, the sections were stained with hematoxylin and eosin (H&E) and imaged using an automatic slide scanning system (AxioScan.Z1, Zeiss, Germany) at 10× magnification for egg-granuloma observation.

### In vivo administration protocol

To deplete macrophages, *M. fortis* received i.p. injections of clodronate liposomes (350 µL per animal; LIPOSOMA, Netherlands) or PBS liposomes every 3 days. To block CR3 function, per *M. fortis* was i.p. injected with 1 mg of CD11b mAb (BioXcell, USA) every 3 days. To achieve maximum reduction of serum complement, *M. fortis* received an i.p. injection of CVF (1 g/kg body weight) or vehicle control solution (PBS) of the same volume every 3 days. To inhibit macrophage-mediated trogocytosis, *M. fortis* i.p. received 1.65 mg/kg/day PP2 or vehicle control (5% DMSO, 45% PEG300, 50% PBS) once daily. To inhibit the Ca^2+^ signal pathway, *M. fortis* i.p. received 2 mg/kg/day 2-APB or vehicle control (PBS) once daily. To block NFAT function, *M. fortis* i.p. received 3 mg/kg/day FK506 or vehicle control (corn oil) once daily. All drugs were administered on the day after *M. fortis* infected with 200 *S. japonicum* cercariae. After treatment, the *M. fortis* were sacrificed 13 days after infection to evaluate cell adherence and schistosomula killing. To knock down Nfatc2 in *M. fortis*, *M. fortis* were administered 100 μL AAV8-shRNA-NC (non-targeting control) or AAV8-shRNA-NFATc2 (1.5 × 10^12^ viral genomes/mL; OBiO Technology Corp., Ltd, Shanghai, China) through tail vein injection, and then infected with *S. japonicum* 2 weeks later. The *M. fortis* were sacrificed 11 days after infection to evaluate cell adherence and schistosomula killing.

### TEM analysis

Transmission electron microscopy analysis was performed as previously described^72^. In brief, the samples were fixed in 2.5% glutaraldehyde in 0.2 M cacodylate buffer, pH 7.4 for over 24 h. After being washed and dehydrated in ethanol gradient, the samples were embedded in araldite. Ultrathin sections were obtained using an ultra-microtome and collected on 200-mesh grids, counterstained with 1% methanolic uranyl acetate and Reynold’s solution of lead citrate. The sections were observed in a Hitachi H-300 transmission electron microscope (Japan).

### Trogocytosis fluorescence assay

The transfer of membrane from schistosomula to macrophages was detected by laser scanning confocal microscope. Schistosomula were labeled with Vybrant® CFDA SE Cell Tracer (2 µM, ThermoFisher, USA) for 15 min at 37 °C, and after discarding the staining solution, incubated in complete medium for 30 min. After washing with PBS, the parasites were incubated together with macrophages of mice or *M. fortis* labeled with Cell Tracker™ fluorescent probes (8 µM, ThermoFisher, USA) at an E:T ratio of 2000:1 in the presence of 5% mouse or *M. fortis* serum. In the *M. fortis* macrophage culture system, PP2 (20 µM), cytochalasin D (10 µM) or FK506 (10 ng/mL) were additionally added according to the purpose of the experiments. After the incubation for 12 h, samples were fixed with stop buffer containing 0.4% PFA and observed on a Nikon A1R N-SIM N-STORM microscope (Nikon, Japan). The images were acquired by Z-stack spanning. Data were then deconvolved using *NIS*-*Elements* software (Nikon, Japan), processed and a 3D reconstruction carried out. The percentage of cells positive for trogocytosis and the mean fluorescent intensity/single cell were analyzed using Imaris 8.4 software *(*Bitplane*)*.

### Immunofluorescence detection

PFA-fixed 13-day schistosomula collected from *M. fortis* and the schistosomula co-cultured with *M. fortis* macrophages in vitro were blocked with 5% bovine serum albumin in PBS for 30 min after being washed with 0.01% Tween 20 in PBS. Then the parasites were incubated with anti-mouse CD11b (eBioscience, CA) and anti-complement C3b/iC3b (BioLegend, USA) primary antibodies overnight at 4 °C. The samples were then incubated with *Alexa Fluor*® 488 or *Alexa Fluor* 596-conjugated secondary antibody. The nucleus was stained with DAPI (Thermo Fisher Scientific, USA). Immunofluorescence signals were acquired by LSM780 laser scanning confocal microscope (Zeiss, Germany) observations.

### Flow cytometry analysis

Liver leukocytes of *M. fortis* were prepared by Percoll gradient centrifugation after being dispersed through a 70-μm nylon strainer. The cell precipitates were washed twice and suspended in PBS containing 0.1% BSA and 0.05% sodium azide. A total of 2 × 10^6^ cells per 100 μL were incubated with CD11b-FITC (eBioscience, CA) for 30 min at 4 °C in the dark. The expression of CD11b was analyzed on a CytoFLEX flow cytometer (Beckman Coulter, USA) using CytExpert software (Treestar, CA).

### In vitro monitoring the survival rate of 7-day and 14-day-old schistosomula

After harvesting the *S. japonicum* from infected mice and *M. fortis* at 7 days and 14 days post-infection, the worms were washed 4 times in 37 °C RPMI-1640 medium (containing 1% HEPES buffer, 2% penicillin/streptomycin and 2% _L_-glutamine) and then cultured in RPMI-1640 complete medium (supplemented with 10% FBS, 1% HEPES buffer, 1% sodium pyruvate, 1% glucose, 1% nonessential amino acids, 2% penicillin/streptomycin and 2% _L_-glutamine) at 37 °C and 5% CO_2_. Half the volume of the culture was refreshed every 2 days with fresh medium^75^. In the process of culture, the number of dead worms was counted daily.

### Analysis of cytosolic free Ca^2+^ concentration ([Ca^2+^]c)

Changes in [Ca^2+^]c were analyzed by confocal microscopy. Macrophages exposed to mouse or *M. fortis* serum (5%) or 2-APB (10 µM) treatment under regular RPMI-1640 medium (containing Ca^2+^) were stimulated with SWA (10 μg/mL) in triplicate and incubated at 37 °C in a humidified atmosphere of 5% CO_2_ for 6 h. The cells were washed with PBS and then loaded with 4 μmol/L Fluo-4-AM for 40 min in dark at 37 °C. After washing with PBS, cells were further incubated at 37 °C for 30 min and imaged on a laser scanning confocal microscope (LSM780, Zeiss, Germany). Fluo-4 was excited at 488 nm using an argon laser line, and emitted fluorescence was collected through a 530 nm band-pass filter. [Ca^2+^]c was calculated by the mean fluorescent intensity and analyzed using Image-Pro Plus 6.0 software.

### RNA extraction and real-time PCR

RNA extraction and determination of mRNA expression were performed as described previously^76^. Briefly, total RNA was extracted from *M. fortis* macrophages or liver tissues using TRIzol and purified using RNeasy Mini Kit (Qiagen, USA) following the manufacturer’s instructions. Real-time PCR for quantitative mRNA expression analyses was performed on a *CFX96* RealTime PCR System (Bio-Rad, USA) using the SYBR Green Master Mix kit (Takara, Japan). Relative expression was normalized to the internal gene GAPDH and calculated using the 2^−ΔΔCT^ method. The primers were listed in Supplementary Table S1.

### Statistical analysis

All graphs and statistical analyses were performed using GraphPad Prism 7.0 (GraphPad Software Inc., San Diego, CA, USA). All data are shown as mean ± SEM and as a single value. Data were analyzed by Student’s unpaired *t*-test with Welch’s correction for two-group comparisons and by One-way ANOVA with Tukey’s multiple comparisons for comparisons of three or more groups. Pearson correlation analysis was used for correlation. *P-*values < 0.05 were considered to be statistically significant.

### Supplementary information

Supplementary Information

Supplementary Video S1

Supplementary Video S2

Supplementary Video S3

Supplementary Video S4

Supplementary Video S5

Supplementary Video S6

Supplementary Video S7

Supplementary Video S8

Supplementary Video S9

## Data Availability

Raw RNA-Seq sequencing data have been submitted to the NCBI Sequence Read Archive under BioProject accession number PRJNA860756. All other data supporting the findings of this study are available within the article and its supplementary files. Additional data related to this paper may be requested from the authors. Source data are provided with this paper.
