# Supplementary material for: Macrophage-mediated trogocytosis contributes to destroying human schistosomes in a non-susceptible rodent host, Microtus fortis
Source: Cell Discov. 2023 Oct 5;9:101. doi: 10.1038/s41421-023-00603-6 (PMC10550985; doi:10.1038/s41421-023-00603-6)
Supplement: Supplementary file 1 — Supplementary Information [file 41421_2023_603_MOESM1_ESM.pdf]

Supplementary information

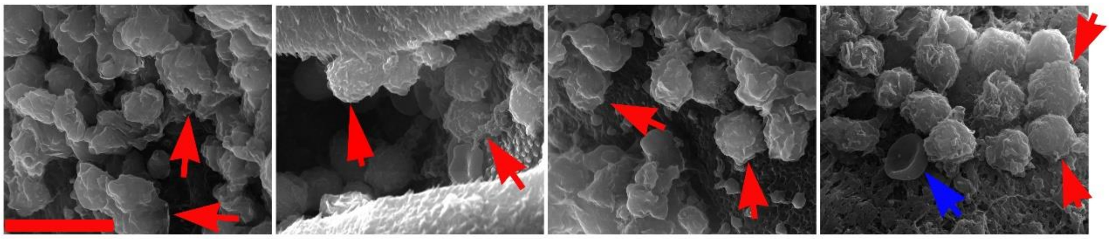

Fig. S1 SEM showing the morphology of the cells adhered on the surface of *S. japonicum* in *M. fortis*. Scale-bars: 10 µm. The red arrows indicate leukocytes, and the blue arrow indicates an erythrocyte.

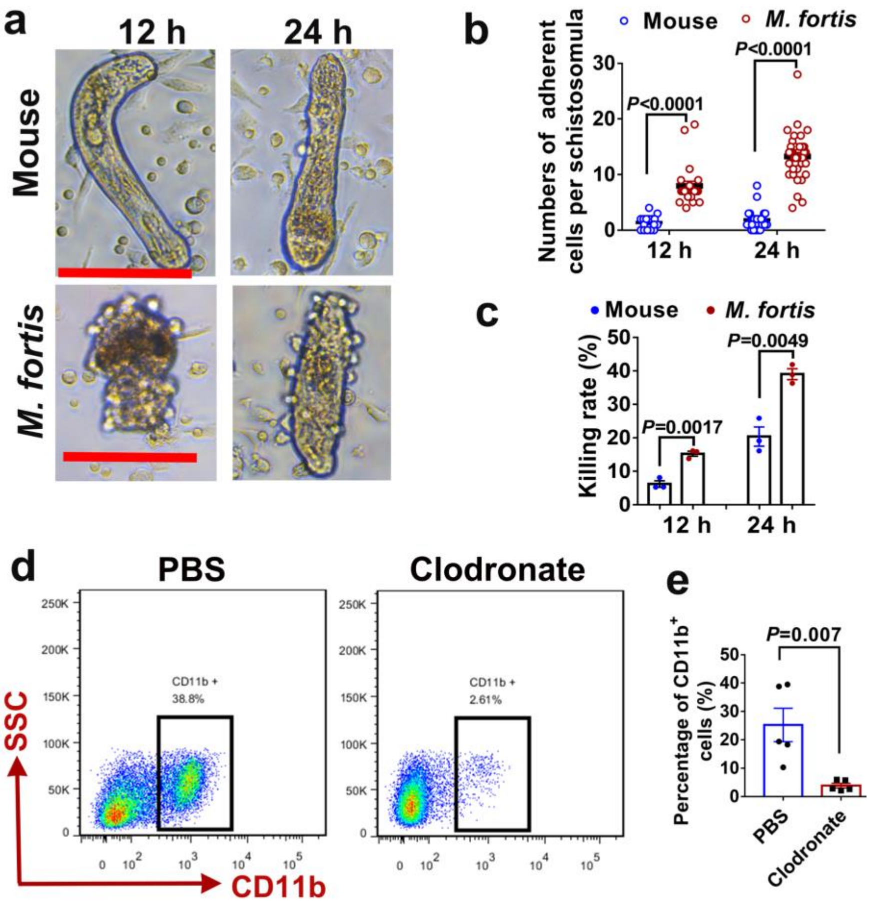

Fig. S2 *M. fortis* macrophages mediate adherence and killing of schistosomes. a *M. fortis* macrophages mediate adherence and killing of *S. mansoni* in a similar manner to *S. japonicum.* Comparison of cell adherence to the schistosomula of *S. mansoni* after incubation of the schistosomula with macrophages from BALB/c mice or *M. fortis* for 12 and 24 h in RPMI-1640 medium supplemented with 10% non-heat-inactivated FBS in vitro. Scale-bars: 100 µm. **b** The number of cells adhering to the

schistosomula of *S. mansoni*. **c** The *S. mansoni* schistosomula killing rates after co-culture with macrophages from BALB/c mice or *M. fortis* for 12 and 24 h. The data are expressed as the mean ± SEM of three animals per group. Data are representative of three independent experiments. **d** Flow cytometry analysis shows the percentage of CD11b$^+$ macrophages in the livers of infected *M. fortis* at 28-days post-infection treated with PBS- or clodronate- liposomes, indicating that the clodronate liposome treatment effectively depleted monocyte-macrophage cell populations in *M. fortis*. The CD11b$^+$ cells were gated from the monocyte population. **e** The frequency data of CD11b$^+$ cells from the flow cytometry presented in **d**. n = 5 *M. fortis* per group. Data shown are mean ± SEM and repeated twice with similar results.

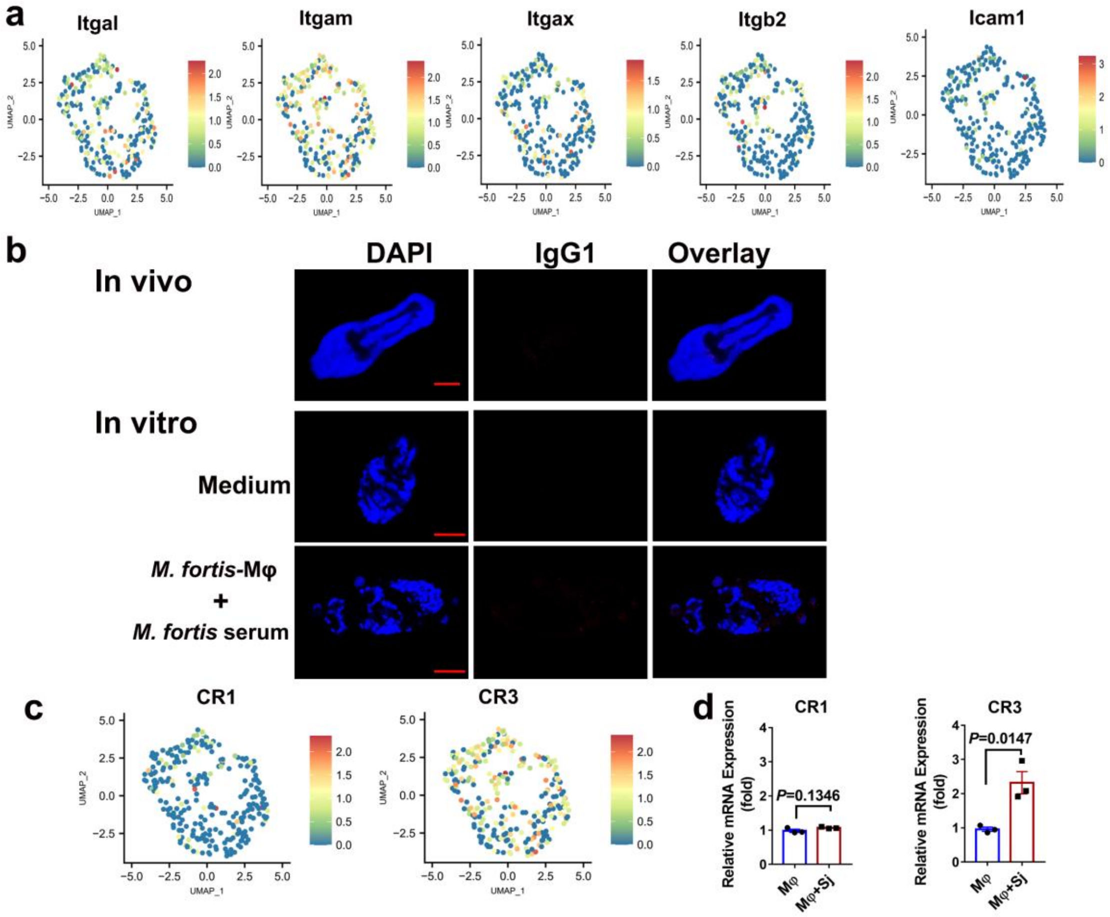

Fig. S3 ***M. fortis*** **macrophages mediated adherence and killing of** ***S. japonicum*** **required Complement C3 and its receptor CR3. a** UMAP maps show the expression of adhesion molecules in scRNA-seq analysis. CD11a (*Itgal*); CD11b (*Itgam*); CD11c (*Itgax*); CD18 (*Itgb2*); ICAM1 (*Icam1*). Data are shown as normalized transcript counts on a color-coded logarithmic scale. **b** The IgG1 isotype control of immunofluorescence detecting C3b/iC3b on the surface of *S. japonicum* collected from *M. fortis* on 13 days after infection (In vivo, scale-bars: 50 μm) and the schistosomula co-cultured with *M. fortis* macrophages in the presence of 5% *M. fortis*

serum or culture medium alone in vitro (scale-bars: 20 μm). The nucleus was stained with DAPI (blue). **c** UMAP maps show the expression of CR1 (*CD35*) and CR3 (*Itgam*) in scRNA-seq analysis. **d** Comparison of the relative expression of CR1 and CR3 (*Itgam*) in *M. fortis* macrophages after incubation of schistosomula of *S. japonicum* for 6 h.

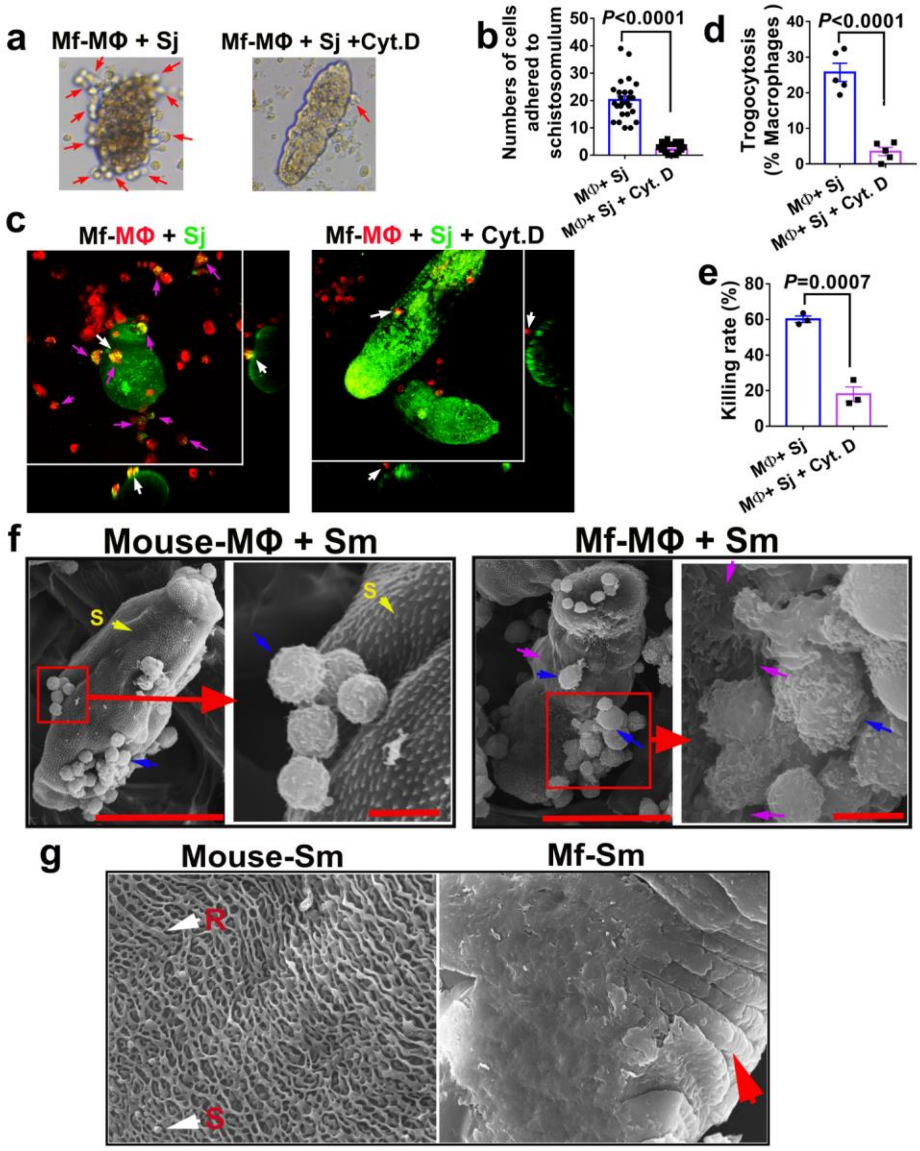

Fig. S4 Trogocytosis participated in destroying schistosomes by *M. fortis* macrophages. a-e Analysing the effects of *M. fortis* macrophages on cell adhesion to the schistosomula (a and b), trogocytosis (c and d) and the schistosomula killing rate (e) in the presence of 10 μM cytochalasin D (Cyt. D) in vitro. The red arrows indicate macrophage adherence to schistosomula. Magenta arrows indicate the macrophages with positive trogocytosis. White arrows indicate the selected typical cell that adhered to the schistosomula. $n = 3$ *M. fortis*. Data represent mean ± SEM and repeated twice with similar results. **f** SEM images of the trogocytic outcomes on the surface of

schistosomula (*S. mansoni*) by macrophages (blue arrows) isolated from mouse (Mouse- MΦ) and *M. fortis* (Mf- MΦ) after incubation for 16 h in the presence of 5% serum. S, spine; Sm, *S. mansoni*. Yellow arrows show the remaining spines in the epidermis of schistosomula; magenta arrows show the exposed muscle layer through the bitten off epidermis of schistosomula. The whole worm was photographed at 3500× (Scale-bars: 40 µm); local parts of the worm were photographed at 15,000× (Scale-bars: 5 µm). **g** SEM images of the trogocytic outcomes of *S. mansoni* by leukocytes in the mouse and *M. fortis* on 21 days post-infection. Sm, *S. mansoni;* R, ridge; S, spine. Red arrows show the exposed muscle layer after the epidermis exfoliated. Similar results were obtained with experiments repeated twice.

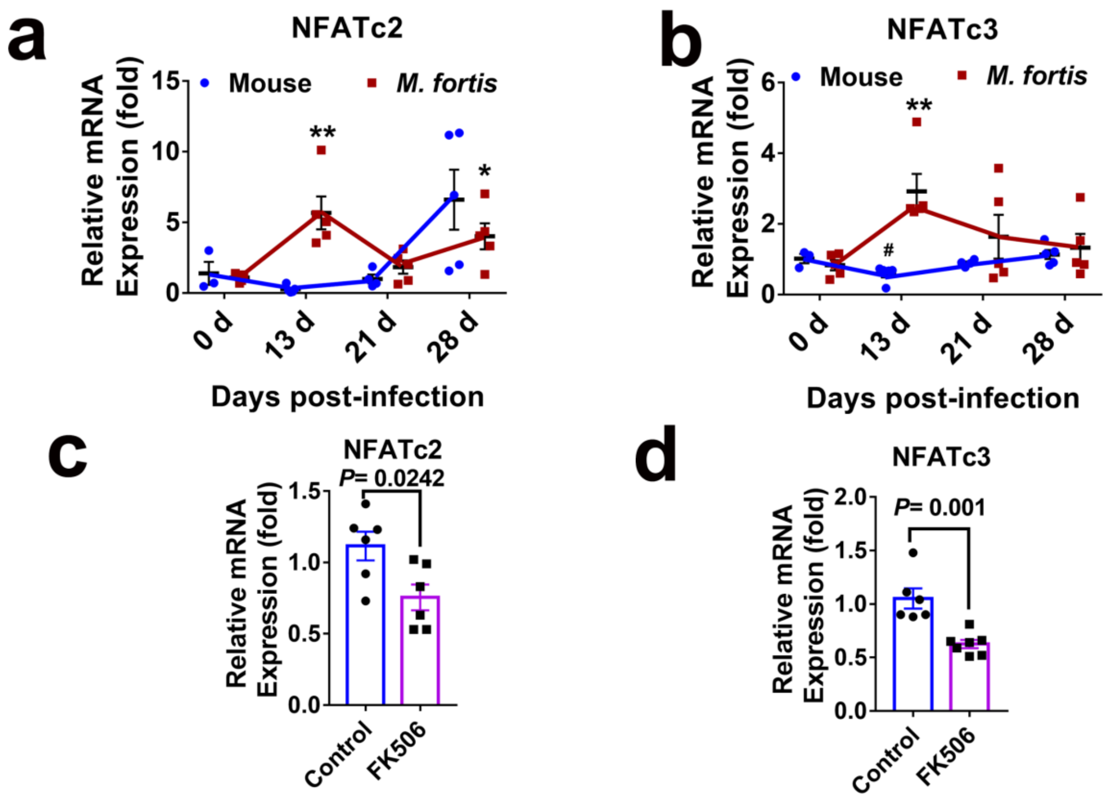

Fig. S5 The expression of NFATc2 and NFATc3 in the liver of mice and *M. fortis*. **a-b** Comparison of the relative expression of NFATc2 and NFATc3 in the liver of mice and *M. fortis* during different periods of *S. japonicum* infection. $^{**}P < 0.01$, compared with the *M. fortis* group on 0 day; $^{\#}P < 0.05$, compared with the mouse group on 0 day. **c-d** The NFAT inhibitor FK506 efficiently diminished the expression of NFATc2 and NFATc3 in the liver of *M. fortis* infected with *S. japonicum.* All data are expressed as the mean ± SEM. All data are expressed as the mean ± SEM of 5-6 animals per group. Data are representative of at least three independent experiments.

Video. S1 Dynamic detection of cell adhesion to schistosomulum of *S. japonicum* and worm vitality by co-culture of mouse macrophages and schistosomula from 0 to 12 h in vitro.

Video. S2 Dynamic detection of cell adhesion to schistosomulum of *S. japonicum* and worm vitality by co-culture of *M. fortis* macrophages and schistosomula from 0 to 12 h in vitro.

Video. S3 Dynamic detection of cell adhesion to schistosomulum and worm vitality by co-culture of *M. fortis* macrophages and schistosomula in the presence of normal *M. fortis* serum from 0 to 12 h in vitro.

Video. S4 Dynamic detection of cell adhesion to schistosomulum and worm vitality by co-culture of *M. fortis* macrophages and schistosomula in the presence of C3-inactivated serum of *M. fortis* with CVF treatment from 0 to 12 h in vitro.

Video. S5 Dynamic detection of cell adhesion to schistosomulum and worm vitality by co-culture of *M. fortis* macrophages and schistosomula in the presence of normal *M. fortis* serum and CD11b mAb from 0 to 12 h in vitro.

Video. S6 3D image shows BALB/c mouse macrophages (red) with trogocytic uptake of schistosomula membrane material (green) after co-culturing for 12 h.

Video. S7 3D image shows *M. fortis* macrophages (red) with trogocytic uptake of schistosomula membrane material (green) after co-culturing for 12 h.

Video. S8 3D image shows *M. fortis* macrophages (red) with trogocytic uptake of schistosomula membrane material (green) after treatment with the trogocytosis inhibitor PP2.

Video. S9 Dynamic detection of cell adhesion to schistosomulum and worm vitality by co-culture of *M. fortis* macrophages and schistosomula in the presence of normal *M. fortis* serum and 2-APB from 0 to 12 h in vitro.

Supplementary Table S1. Primer sequences used in the study

Species	Gene	Foward (5'-3')	Reverse (5'-3')
M. fortis	GAPDH	CCACCCATGGCAAGTTCAAA	ATCTCGCTCCTGGAAGATGG
	CR1	TGCCATTCTACTGGGCTTGA	GAAGGAACAGCAGCAGGATG
	Itgam	CCACTTATTGTGGGCAGCTC	TATTGCCGCTTGAAGAAGCC
	Nfatc2	TCACTACTCACCCACCAACC	TGGTAGCTCTGTGGTTCAGG
	Nfatc3	TCAGCTGCAGTCTATGCCTT	GACCTCCTTGGCCTGTACTT
**Murine**	GAPDH	AACGGATTTGGCCGTATTGG	CATTCTCGGCCTTGACTGTG
	Nfatc2	CTGGGCAGAATTCTCGTGTG	GGCATTGCTCCAGTCAGAAG
	Nfatc3	AGGACTCCAGTTGAGAAGGC	GTGTGGAAGGACAGGTCTGA
